# Mast Cell Phenotypic Heterogeneity Impacts the Interplay with Pathogenic *Salmonella* Typhimurium Bacteria

**DOI:** 10.1002/eji.70040

**Published:** 2025-08-21

**Authors:** Christopher von Beek, Grisna I. Prensa, Julia H. M. Andersson, Gunnar Pejler, Mikael E. Sellin

**Affiliations:** ^1^ Department of Medical Biochemistry and Microbiology Uppsala University Uppsala Sweden; ^2^ Science for Life Laboratory Uppsala Sweden

**Keywords:** cytokine response, intracellular bacteria, mast cells, *Salmonella*, serglycin

## Abstract

Mast cells (MCs) lodge within barrier tissues and respond to infectious microbes. Recent work demonstrated that MCs differentiate their cytokine response to extracellular versus invasive Gram‐negative enterobacteria by a two‐step activation mechanism that integrates Toll‐like‐receptor (TLR) sensing with signals elicited by type‐III‐secretion‐system (TTSS) effectors during bacterial invasion. However, multiple MC subtypes exist, and it remains unclear how their phenotypic heterogeneity impacts microbial interactions. We find that murine MCs maintained in IL‐3, or differentiated toward a connective‐tissue phenotype (CT‐MCs), respond potently to the enteropathogen *Salmonella enterica* Typhimurium (*S*.Tm) through two‐step activation, with the TLR component explained by functional TLR4 and TLR2. By contrast, murine mucosal mast cells (M‐MCs) express insignificant levels of these TLRs, therefore being unresponsive to extracellular *S*.Tm, but still mounting a response to invasive bacteria. Following invasion, MC granule maintenance by serglycin restricts *S*.Tm vacuolar and cytosolic colonization. Notably, this has no impact on the cytokine release from infected MCs, thus uncoupling *S*.Tm´s intracellular life‐cycle from the MC cytokine response. Finally, human LUVA MCs employ a variant of two‐step activation where TLR2/6 signaling combines with the TTSS‐elicited signals. Together, this study explains how MC subtypes can respond differently to *S*.Tm‐infection depending on their TLR expression and granule features.

## Graphical abstract

Mast cell phenotypic features influence anti‐bacterial response. Connective tissue‐like mast cells sense *Salmonella* through combined Toll‐like‐receptor (TLR) signaling and recognition of Type‐III‐secretion‐system mediated invasion. By contrast, due to low TLR expression, mucosal‐like mast cells fail to respond to extracellular *Salmonella*, but their abundant granules limit intracellular colonization by invasive bacteria.

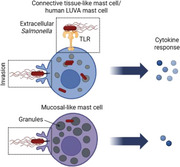

**Abbreviations**
4KO: knockout of Mcpt4, Mcpt5, Mcpt6, and CPA3
BMMC: bone marrow‐derived mast cell
CPA3: carboxypeptidase A3
CT‐BMMC: connective tissue‐like bone marrow‐derived mast cell
DEG: differentially expressed gene
M‐BMMC: mucosal‐like bone marrow‐derived mast cell
MC: mast cell
Mcpt: mast cell protease
MOI: multiplicity of infection (bacteria per host cell)
p.i.: (time) postinfection
PCMC: peritoneal cell‐derived mast cell
PRR: pattern recognition receptor
*S*.Tm: *Salmonella enterica* serovar Typhimurium
SG: Serglycin
TEM: transmission electron microscopy
TLR: Toll‐like receptor
TTSS‐1: type‐three‐secretion‐system 1

## 1 Introduction

Mast cells (MCs) are granulated innate immune cells that respond to a variety of stimuli and can stem from both a hematopoietic and embryonic origin. While tissue‐resident MCs develop during embryogenesis, bone marrow progenitor‐derived MCs can be recruited from the peripheral blood to mature at tissue sites [1]. In adult mammals, MCs are found within nearly all tissues of the body, where their phenotype is influenced by niche‐specific cytokines and growth factors specific for that environment [1, 2]. A combination of origin and tissue site‐specific cues, hence, gives rise to distinct MC subtypes. In mice, these are traditionally classified as either connective tissue‐type MCs (CT‐MCs) or mucosal‐type MCs (M‐MCs) [1]. CT‐MCs are best characterized in the skin and peritoneal cavity and express a wide range of MC‐specific proteases, including the MC proteases 4–7 (Mcpt4, 5, 6, 7) as well as carboxypeptidase A3 (CPA3) [3]. M‐MCs, on the other hand, are predominantly recruited from the blood into mucosal layers, including the lung and intestine, and express Mcpt1 and Mcpt2 [3]. Complex barrier tissues such as the intestinal wall harbor both M‐MC and CT‐MC subtypes with differential localization patterns, and with total MC numbers and the M‐MC/CT‐MC ratio varying across physiological and inflammatory states [4, 5]. In humans, MC subtypes are less clear‐cut, and classification has traditionally relied on assessment of protease content, separating MC populations expressing tryptase (MC_T_) from those expressing tryptase and chymase (MC_TC_) [1, 3]. Classification based on proteases alone, however, is unlikely to cover the full heterogeneity of MCs. This was demonstrated recently by more diverse transcriptional profiles in single‐cell RNA sequencing data [4, 5].

MCs located within barrier tissues come in close contact with pathogenic microbes and have been shown to contribute to the defense against a variety of infectious agents [6, 7, 8, 9], including Gram‐negative enterobacteria [10, 11, 12]. One such enterobacterium is *Salmonella enterica* serovar Typhimurium (*S*.Tm), a flagellated rod‐shaped pathogen and leading cause of gut inflammatory disease worldwide [13]. *S*.Tm attacks the gut mucosa and triggers its own uptake by injecting effectors into the host cell cytosol through a Type‐III secretion system (TTSS‐1). Upon entering the intracellular environment, S.Tm takes up residence in a modified endosomal vesicle, known as the *Salmonella*‐containing vacuole [14]. Although mainly characterized as a vacuolar pathogen, in a subset of infected cells, *S*.Tm lyses the vacuolar membrane and proliferates freely in the cytosol [14]. This dual intracellular lifestyle involves a trade‐off with better access to nutrients in the cytosol, but a higher risk for the pathogen of being detected by the host‐cell pathogen recognition receptors (PRRs) [15, 16]. Moreover, TTSS‐1 expression is heterogenous within the *S*.Tm population [17, 18], causing a large fraction of the bacteria to remain in an extracellular state during an ongoing infection.

Our recent work has demonstrated that *S*.Tm in the gut indeed encounter MCs in both the inflamed lumen and in the superficial intestinal mucosa, and that *S*.Tm can efficiently invade MCs through TTSS‐1 effector translocation [11]. Intriguingly, MCs were found capable of differentiating between extracellular and invasive *S*.Tm through a “two‐step activation mechanism” that combines PRR sensing with effector‐triggered immunity. Specifically, murine bone‐marrow‐derived mast cells (BMMCs) employ the PRR Toll‐like receptor 4 (TLR4) to recognize extracellular and invasive Gram‐negative enterobacteria. *S*.Tm invades MCs and in the process translocates the TTSS‐1 effectors SopB, SopE, and SopE2, which fuel a second set of signals that involve Akt phosphorylation. By differentiating between these one versus two types of input, MCs were found to produce modest levels of immunostimulatory cytokines (e.g., IL‐6 and TNF) when encountering extracellular enterobacteria, but vastly higher levels when encountering invasive *S*.Tm [11]. Notably, our avidin staining and mRNA profiling [11], as well as work by others [4, 5], suggest that the MC populations encountered by *S*.Tm in the mammalian gut are highly diverse. However, it has remained unclear how MC subtype heterogeneity affects the interplay with infecting microbes. Here, we elucidate how differential granulation and PRR expression profiles of murine CT‐MCs, murine M‐MCs, and a human MC model dictate the cellular susceptibility to *S*.Tm infection and the elicited MC cytokine response.

## 2 Results

### 2.1 Murine Connective‐Tissue‐ and Mucosal Mast Cells Respond Distinctly to *Salmonella* Typhimurium

We recently characterized MC responses to *S*.Tm [11], using MC models with connective‐tissue‐like properties such as cultured BMMCs and peritoneal cell‐derived mast cells (PCMCs). Since our prior stainings of *S*.Tm‐infected murine caecum were limited to avidin (preference for CT‐MCs), we here extended that analysis to co‐staining for Mcpt1 (marker for M‐MCs). *S*.Tm was, as expected, still present in the caecal lumen and tissue at a chronic infection stage of 42 days p.i. (Figure S1A). Mcpt1 staining demonstrated the presence of M‐MCs, which appeared exclusively in the mucosa, while avidin^+^ CT‐MCs were present in both mucosa and submucosa (Figure S1B). Unlike in, for example, nematode infection [19], *S*.Tm challenge did not lead to a striking increase in M‐MC numbers. Instead, broadly comparable frequencies of M‐MCs and CT‐MCs were observed also in the *S*.Tm‐infected mucosa (Figure S1B, [11]).

**FIGURE 1:**
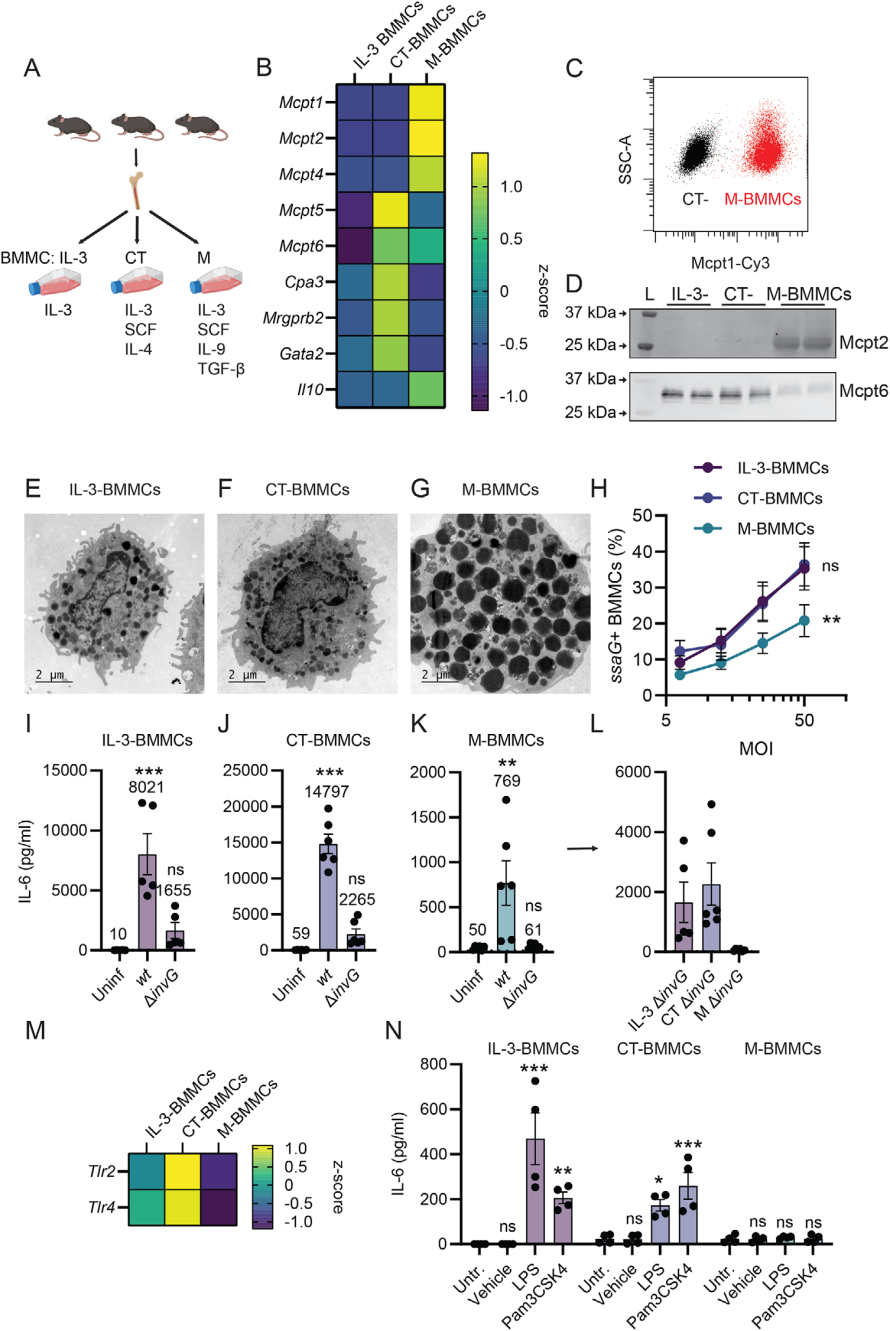
Bone marrow‐derived murine mast cells differentiate into connective‐tissue‐ and mucosal‐like mast cell phenotypes with distinct responses to *S*.Tm infection. (**A**) Differentiation procedure for bone marrow. For every batch of BMMCs, bone marrow from three female mice was pooled and cultured with either IL‐3 (“IL‐3‐BMMCs”), the connective tissue cocktail IL‐3, SCF, and IL‐4 (“CT‐BMMCs”), or the mucosal cocktail of IL‐3, SCF, IL‐9, and TGF‐β (“M‐BMMCs”). (**B**) Transcriptional profile of BMMC subtypes after 5 weeks of culture, shown as z‐scores of 2^−ΔCq^ values relative to *Gapdh* (*n* = 4, from two experiments). (**C**) Representative (*n* = 2, from two experiments) flow cytometry plot for intracellular Mcpt1 staining of CT‐BMMCs (black) and M‐BMMCs (red). (**D**) Representative (*n* = 2, from two experiments) immunoblots of BMMC subtypes for Mcpt2 and Mcpt6 protein (duplicate lanes for one culture shown). Protein from 200,000 cells was loaded to reflect the protease content per cell. For total protein, see immunoblot source data. (**E–G**) Representative (*n* = 2, from two experiments) TEM images of BMMC subtypes (uninfected). (**H**) Percentage of BMMCs harboring vacuolar (*ssaG*‐GFP+) *S*.Tm 4 h p.i. (*n* = 6, from three experiments). (**I–K**) Levels of secreted IL‐6 from the indicated BMMC subtypes infected with MOI 50 of S.Tm^*wt*^ or *S*.Tm^*∆invG*^ for 24 h (**I**: *n* = 5, **J–K**) *n* = 6; all from three experiments. Numbers above bars indicate group means. (**L**) Replotted IL‐6 levels of *S*.Tm^∆^ *^invG^* infected BMMCs. (**M**) *Tlr2* and *Tlr4* transcript levels of BMMC subtypes after 5 weeks of culture, shown as z‐scores of 2^−ΔCq^ values relative to *Gapdh* (*n* = 4, from two experiments). (**N**) Levels of secreted IL‐6 from BMMC subtypes that were either left untreated (Untr.) or treated with 0.5 µg/mL LPS, 10 µg/mL Pam3CSK4, or its vehicle (0.5% ethanol) for 4 h (*n* = 4, from two experiments). For (**H–L**) and (**N**), means ± SEM are shown. Except in *N*, all data are based on two separate BMMC cultures per culture condition. (**H**) IL‐3‐BMMCs were compared with other groups by two‐way ANOVA with Dunnett's post hoc test. (**I–L**, **N**) Uninfected or untreated MCs were used for statistical comparisons by one‐way ANOVA and Dunnett's post hoc test to all other groups. *p < 0.05; **p < 0.01; ***p < 0.001; ns—nonsignificant.

To examine how the two‐step MC activation mechanism by enterobacteria applies across different MC subtypes, we next explored differentially cultured mouse MC models. Based on established protocols [19, 20, 21, 22, 23], we utilized MC subtype‐specific cytokine cocktails to generate connective‐tissue‐like bone‐marrow‐derived mast cells (CT‐BMMCs) and mucosal‐like bone‐marrow‐derived mast cells (M‐BMMCs). Pooled bone marrow samples were cultured in the presence of IL‐3, SCF, and IL‐4 (for CT‐BMMC differentiation), or IL‐3, SCF, IL‐9, and TGF‐β (for M‐BMMC differentiation), and compared with our standard “IL‐3‐BMMC” protocol (Figure 1A). After 4 weeks of culture, all BMMC subtypes contained >95% FcεRI^+^ CD117^+^ cells (Figure S2A,B). Flow cytometry further revealed a similar scatter profile for IL‐3‐ and CT‐BMMCs, while M‐BMMCs appeared more diverse and more granulated, as judged by side scatter (Figure S2A). An analysis of key MC transcript levels after 5 weeks of culture showed that M‐BMMCs indeed upregulated the mucosal MC proteases *Mcpt1* and *Mcpt2* as well as *Il10*, while CT‐BMMCs featured higher transcript levels of, for example, *Mcpt5* and *Mrgprb2* (Figure 1B). Although in prior work [22, 24], 1–2 ng/mL IL‐4 has often been used, we found that 10 ng/mL IL‐4 added directly from the start of culturing was required for proper induction of CT‐MC transcripts (Figure S2C). At the protein level, >99% of M‐BMMCs contained Mcpt1 (Figure 1C), expressed Mcpt2, and had markedly lower levels of Mcpt6 compared with IL‐3‐ and CT‐BMMCs (Figure 1D). In addition, M‐BMMCs contained more CD63 on their surface during steady‐state, as well as upon activation with the calcium ionophore A23187 (Figure S2D). Strikingly, while IL‐3‐ and CT‐BMMCs appeared weakly granulated, transmission electron microscopy (TEM) analysis revealed dramatically higher granulation of M‐BMMCs (Figure 1E–G). Hence, differential culturing generates phenotypically distinct CT‐BMMC and M‐BMMC populations.

After infecting these BMMC subtypes with a *S*.Tm reporter strain (*S*.Tm/p*ssaG*‐GFP), marking MCs with vacuolar *S*.Tm inside them, IL‐3‐ and CT‐BMMCs harbored similar levels of vacuolar *S*.Tm at 4 h p.i., whereas this frequency was modestly diminished in M‐BMMCs (Figure 1H). Since we have previously shown that IL‐3‐BMMCs do not degranulate when infected with *S*.Tm [11], we validated these findings also in the more heavily granulated M‐BMMC model. For this purpose, we measured Mcpt1 release into the supernatants 4 h p.i. by ELISA, but did not find any differences between uninfected and infected cells (Figure S2E). Notably, the baseline Mcpt1 levels were relatively high, with ca 2000–3000 ng/mL Mcpt1 in the cell culture supernatants from M‐BMMCs (Figure S2E). In contrast, CT‐BMMCs did not show any Mcpt1 secretion under any condition (<0.235 ng/mL). To investigate whether our model of two‐step MC activation by *S*.Tm applies across BMMC subtypes, we measured the levels of secreted IL‐6 24 h p.i. with *S*.Tm. While again, IL‐3‐ (Figure 1I) and CT‐BMMCs (Figure 1J) responded similarly with a modest cytokine response to a noninvasive *S*.Tm^∆^ *^invG^*, and a full‐blown response to *S*.Tm^*wt*^ (consistent with the previous report [11]), M‐BMMCs secreted 10–20 fold lower IL‐6 levels in response to *S*.Tm^*wt*^, and appeared completely unresponsive to *S*.Tm^*∆invG*^ (Figures 1K,L). Prior work had shown that the cytokine response of IL‐3‐BMMCs to *S*.Tm^*∆invG*^ is, to a large extent, Toll‐like receptor 4 (TLR4)‐mediated [11]. Therefore, we assessed *Tlr* transcript levels by qPCR. This revealed that *Tlr2* and *Tlr4* transcript levels were higher in CT‐BMMCs compared with IL‐3‐BMMCs, but M‐BMMCs showed dramatically reduced levels for both mRNAs (Figure 1M). The lack of functional TLR2 and TLR4 in M‐BMMCs was further evident upon treating the cultures with specific agonists (Pam3CSK4 and LPS, respectively). M‐BMMCs were completely unresponsive to either of these ligands, by contrast to IL3‐ and CT‐BMMCs (Figure 1N). TLR4 expression was accompanied by IL‐6 secretion upon *S*.Tm infection, although the pure LPS response of IL‐3‐BMMCs was overall more variable than in CT‐BMMCs, making it difficult to compare subtle differences between these MC subtypes (Figure S2G). As an additional extension, we measured the levels of secreted IL‐13, which is also included in the broad MC cytokine response to *S*.Tm [11]. As shown in Figure S2F, IL‐13 secretion was lower and more variable than for IL‐6, but showed a similar induction pattern, following *S*.Tm challenge. Specifically, M‐BMMCs exhibited reduced IL‐13 induction by *S*.Tm*^wt^* and no detectable response to *S*.Tm^∆^ *^invG^* (Figure S2F).

PCMCs are regarded as a model for mature CT‐MCs. Murine “CT‐PCMCs” again responded to both LPS and Pam3CSK4 by IL‐6 secretion (Figure S2H), which is fully in line with the results from CT‐BMMCs above (Figures 1J,N). We next investigated if the noted TLR‐unresponsive phenotype of M‐BMMCs could be reversed by depriving them of IL‐9 and TGF‐β for 7–10 days subsequent to establishment (Figure S3A). This indeed led to lowered expression of the M‐MC protease transcripts *Mcpt1* and *Mcpt2*, and elevated *Tlr4* transcript levels (Figure S3B), concomitant with the re‐establishment of a detectable cytokine response to both pure LPS and *S*.Tm^*∆invG*^ (Figure S3C). Finally, these reversed M‐BMMCs produced more than fivefold higher levels of secreted IL‐6 upon infection with invasive *S*.Tm^*wt*^ (Figure S3C). We conclude that murine IL‐3‐BMMCs, CT‐BMMCs, and CT‐PCMCs all respond to both invasive and noninvasive *S*.Tm through the previously proposed two‐step activation mechanism that integrates TLR4(2)‐signaling with signals elicited by *S*.Tm TTSS‐1 effectors. By sharp contrast, murine M‐BMMCs fail to detect extracellular *S*.Tm, and respond less vigorously to *S*.Tm^*wt*^, which can be explained by minimal TLR expression.

### 2.2 Impact of Mast Cell Granules on the *Salmonella* Typhimurium Intracellular Niche and Cytokine Response

**FIGURE 2:**
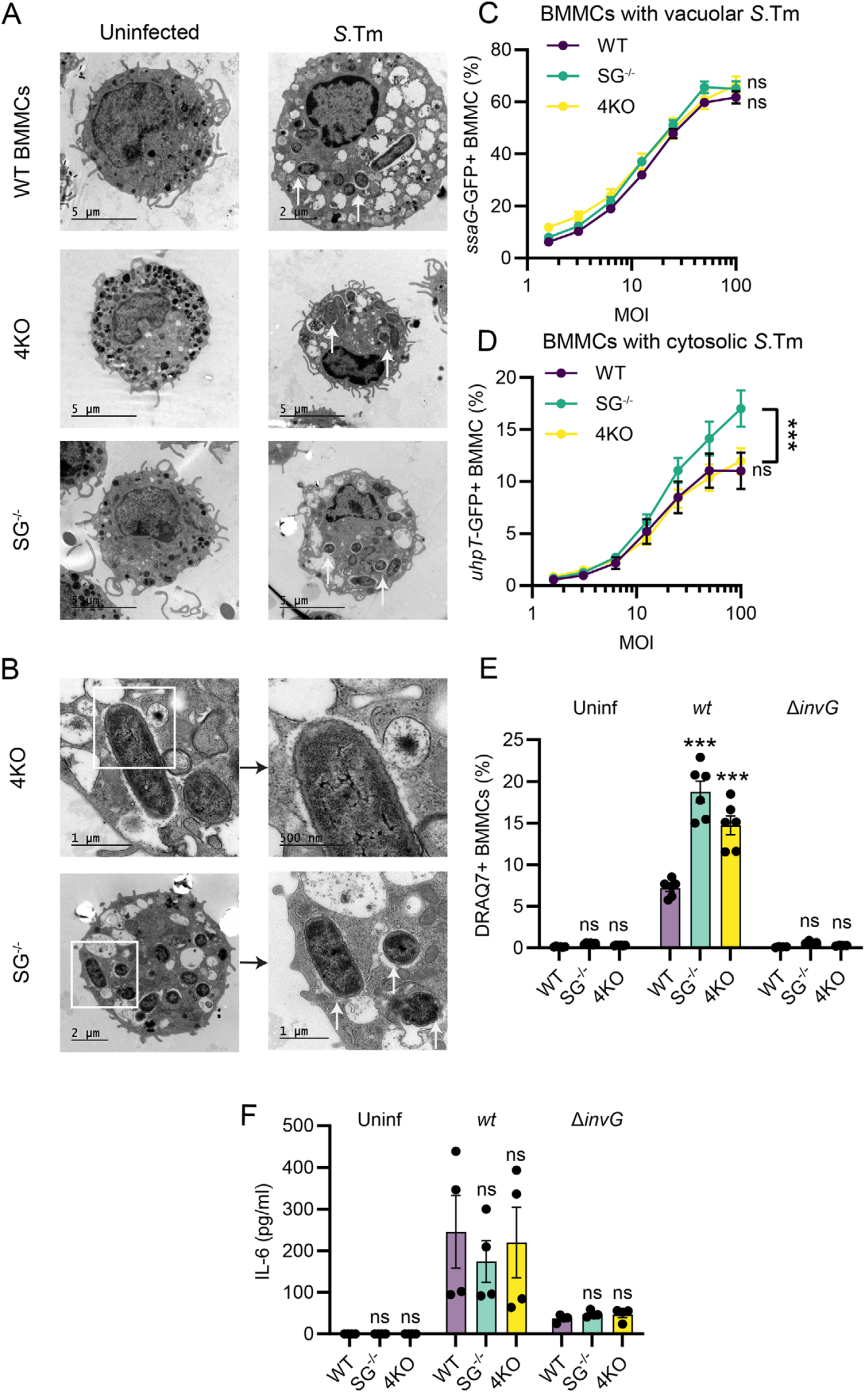
The murine mast cell cytokine response to *S*.Tm appears uncoupled from granular status. (**A**) Representative (from two experiments) TEM images from WT IL‐3‐BMMCs, “4KO” IL‐3‐BMMCs, lacking Mcpt4, Mcpt5, Mcpt6, and CPA3, as well as Serglycin^−/−^ deficient IL‐3‐BMMCs (“SG^−/−^“) uninfected or infected for 4 h with *S*.Tm (white arrows) MOI 50. (**B**) Examples of 4KO and SG^−/−^ IL‐3‐BMMCs where apparent fusion of *S*.Tm‐containing vacuoles and MC granules are observed (enlargements are separate TEM images of the same area in higher magnification). WT images can be found in Figure S4, white arrows indicate *S*.Tm). (**C**) Percentage of BMMCs harboring vacuolar (*ssaG*‐GFP+) *S*.Tm 4 h p.i. (*n* = 8, from four experiments). (**D**) Percentage of BMMCs harboring cytosolic (*uhpT*‐GFP+) *S*.Tm 4 h p.i. (*n* = 8, from four experiments). (**E**) Percentage of DRAQ7+ (dead) BMMCs 4 h p.i. with *S*.Tm*^wt^* or *S*.Tm^∆^ *^invG^* (*n* = 6, 6, 4 (*S*.Tm^∆^ *^invG^*) from three experiments). (**F**) Levels of secreted IL‐6 from BMMCs uninfected or infected with MOI 50 of S.Tm^*wt*^ or *S*.Tm^*∆invG*^ for 4 h (*n* = 4, from two experiments). C‐F show means ± SEM for pooled replicates from two separate BMMC cultures per genotype. WT BMMCs were compared with other groups by two‐way ANOVA with Dunnett's post hoc test. ***p < 0.001; ns, nonsignificant.

MC subsets vary in their degree of granulation. To investigate the intracellular niche of *S*.Tm inside diverse MC subtypes and the role of MC granules during *Salmonella*’s intracellular lifestyle, we performed further TEM analyses on our murine BMMC models at 4 h p.i. In line with the results above (Figure 1E–G), uninfected IL‐3‐ and CT‐BMMCs appeared similar in terms of granule morphology, whereas M‐BMMCs contained more granules, which also appeared more electron dense by comparison (Figure S4A,C,E). In cultures of infected IL‐3‐ and CT‐BMMCs, we frequently observed MCs with *S*.Tm residing within vacuoles close to, or fusing with, granules (Figure S4B,D). Such fusion events were not evident for intracellular *S*.Tm within M‐BMMCs (Figure S4F). The proximity of intracellular *S*.Tm to granule material also did not noticeably affect the bacterial morphology. However, we observed that many granules in infected MCs lost their electron‐dense phenotype (Figure S4B,D,F; Figure 2A). Analysis of cellular Mcpt6 protein content by immunoblotting again revealed no difference between *S*.Tm‐infected and uninfected BMMCs (Figure S4G), further demonstrating the lack of granule release upon infection (see also Figure S2E, [11]). We propose that the infection‐associated change in MC granule density can be explained by fusion with pinocytotic vesicles formed as *S*.Tm elicits membrane ruffling through TTSS‐1 during the invasion step [25].

To probe the impact of MC granules during *S*.Tm infection, we next generated IL‐3‐BMMCs from mice deficient in the dominant granular scaffold glycoprotein serglycin (SG^−/−^), or lacking all the main CT‐type granular proteases (genetically ablated for Mcpt4, Mcpt6, and Cpa3, resulting also in posttranslational absence of Mcpt5; “4KO” [26]). After 4 weeks of culture, WT, SG^−/−^, and 4KO IL‐3‐BMMCs had similar forward‐ and side scatter profiles (Figure S5A) with ∼95% of cells being FcεRI^+^ CD117^+^ (Figure S5B). As expected, 4KO BMMCs lacked Mcpt6, while SG^−/−^ BMMCs featured lower Mcpt6 levels compared with WT cells (Figure S5C). TEM revealed a similar ultrastructure of WT and 4KO BMMCs, distinct from the SG^−/−^, which had brighter, less electron‐dense granules (Figure 2A). For all three genotypes, *S*.Tm were found lodged within the cells, predominantly within vacuolar compartments (Figure 2A). We observed bacteria‐containing vacuoles fusing with granules also in 4KO and SG^−/−^ BMMCs (Figure 2B). Using the two reporter strains *S*.Tm/p*ssaG*‐GFP (vacuolar reporter, used above also) and *S*.Tm/p*uhpT*‐GFP (cytosolic reporter; turns GFP+ upon sensing of glucose‐6‐phosphate), we quantified the MCs containing bacteria in each of these intracellular compartments across MOIs and the three genotypes. By 4 h p.i., there were no differences in the percentage of BMMCs harboring vacuolar *S*.Tm between WT, 4KO, and SG^−/−^ IL‐3‐BMMCs (Figure 2C). However, SG^−/−^ BMMCs harbored cytosolic bacteria at a significantly higher frequency than WT and 4KO cells (Figure 2D). This difference was lost after 24 h with overall lower percentages of BMMCs harboring intracytosolic bacteria (Figure S5D,E). Additionally, we observed higher levels of *S*.Tm‐induced cell death in both 4KO and SG^−/−^ BMMCs compared with WT cells (Figure 2E).

Considering these differences in cytosolic bacterial escape and cell death induction between the BMMC genotypes (Figures 2D,E), we finally probed the cytokine output. Notably, neither IL‐6 (Figure 2F) nor TNF secretion (Figure S5F) differed between *S*.Tm*^wt^*‐infected WT, 4KO, and SG^−/−^ BMMCs. The same held true for *S*.Tm^∆^ *^invG^* infection (Figure 2F; Figure S5F). From these findings, we conclude that granular defects in murine IL‐3‐BMMCs increase the *S*.Tm cytosolic colonization frequency (SG^−/−^) and result in elevated cell death of infected cells (4KO and SG^−/−^). However, this appears to be uncoupled from the MC cytokine response elicited upon *S*.Tm infection.

### 2.3 Serglycin‐Deficient Mucosal Mast Cells Fail to Control *Salmonella* Typhimurium, but Yield Normal Cytokine Levels

As detailed above, we observed differences in the frequency of vacuolar *S*.Tm in murine M‐BMMC compared with IL‐3‐ and CT‐BMMCs (Figure 1H), and a higher frequency of cytosolic *S*.Tm in SG^−/−^ compared with WT IL‐3‐BMMCs (Figure 2D). To further analyze the relationship between MC granulation and *S*.Tm´s intracellular lifestyle, we next established murine M‐BMMC cultures from WT (densely granulated) and SG^−/−^ (disrupted granular integrity) animals. The two cultures were similar with respect to cell size and CD117/FcεRI staining (Figure S6A,B), with WT M‐BMMCs staining slightly stronger for Mcpt1 (Figure S6C). Mcpt2 levels were unchanged by the absence of serglycin, but Mcpt6 (already low in M‐BMMCs; Figure 1D) was essentially lost (Figure S6D).

**FIGURE 3:**
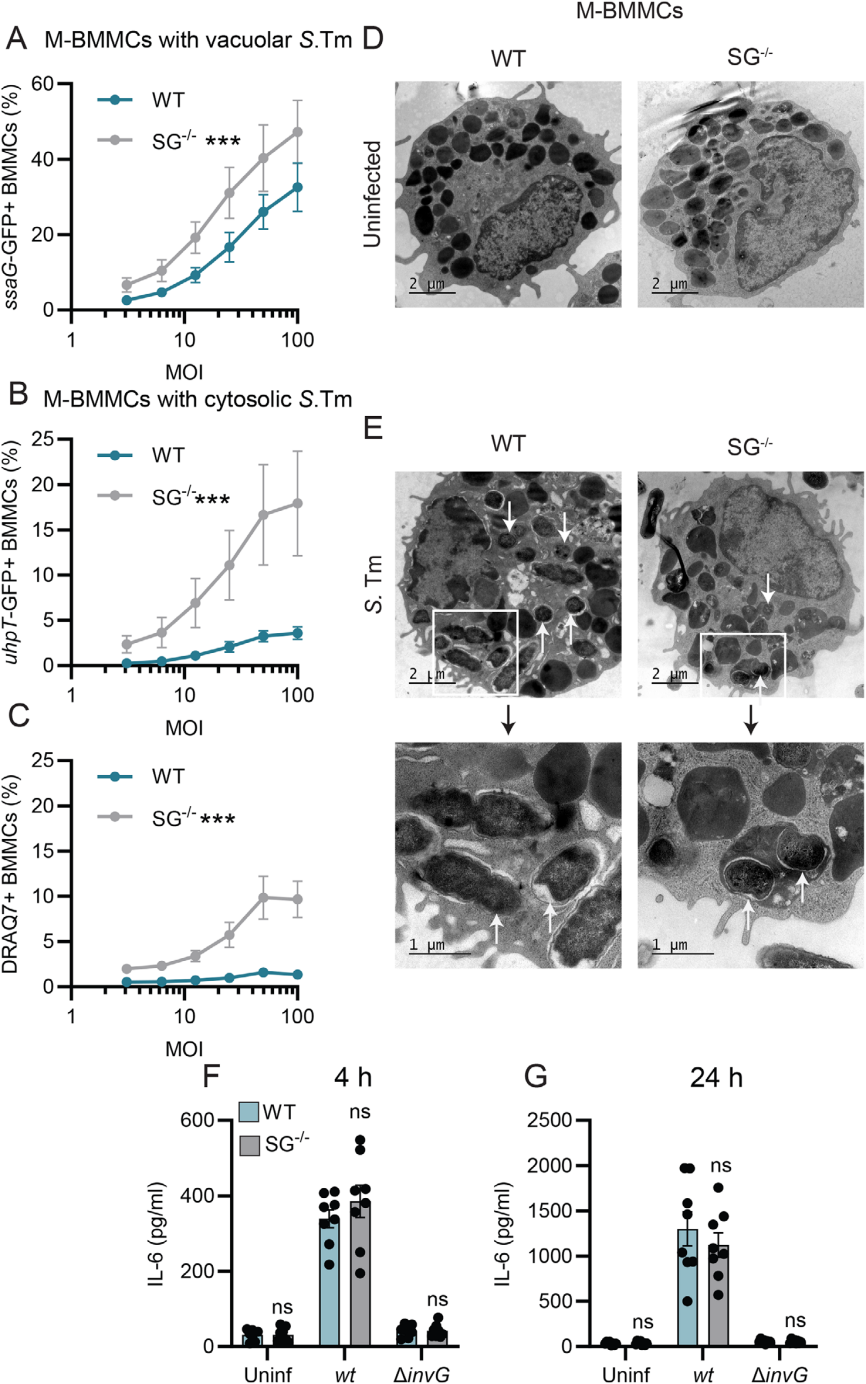
Lack of serglycin affects the intracellular *S*.Tm lifestyle, but not cytokine secretion, in murine mucosal mast cells. (**A**) Percentage of WT and SG^−/−^ M‐BMMCs harboring vacuolar (s*saG*‐GFP+) *S*.Tm 4 h p.i. (*n* = 6, from three experiments). (**B**) Percentage of WT and SG^−/−^ M‐BMMCs harboring cytosolic (*uhpT*‐GFP+) *S*.Tm 4 h p.i. (*n* = 6, from three experiments). (**C**) Percentage of DRAQ7+ (dead) WT and SG^−/−^ M‐BMMCs 4 h p.i. with *S*.Tm^wt^ (*n* = 6, from three experiments). (**D–E**) Representative (for all cells within 1 experiment) TEM images from WT M‐BMMCs and serglycin‐deficient M‐BMMCs (SG^−/−^) uninfected (**D**) or infected (**E**) 4 h with *S*.Tm at MOI 50. Arrows pointing upwards indicate likely vacuolar bacteria with visible space between the bacterium and the cytosol. Arrows pointing downwards indicate likely cytosolic bacteria with no visible space separating the bacterium from the cytosol. Enlargements (separate TEM images of the same area in higher magnification) are shown to highlight multiple *S*.Tm inside electron‐dense MC granules in SG^−/−^cells. (**F, G**) Levels of secreted IL‐6 from WT and SG^−/−^ M‐BMMCs uninfected or infected with MOI 50 of *S*.Tm^*wt*^ or *S*.Tm^*∆invG*^ for 4 h (**F**) and 24 h (**G**) (*n* = 8, from four experiments). (**A–C**) and (**F, G**) show means ± SEM. WT BMMCs were compared with other groups by two‐way ANOVA with Dunnett's post hoc test. ***p < 0.001; ns, nonsignificant.

Using the *S*.Tm/p*ssaG*‐GFP reporter strain for infections, we observed a significantly higher frequency of vacuolar *S*.Tm in SG^−/−^ vs. WT M‐BMMC cultures (Figure 3A). Even more striking, however, was the dramatically elevated levels of cytosolic *S*.Tm (scored by the *S*.Tm/p*uhpT*‐GFP‐reporter strain) (Figure 3B; more than fourfold elevated in SG^−/−^ M‐BMMCs at 4 h p.i.). This phenotype was also evident at 24 h p.i. (Figure S6E), despite a higher frequency of cell death in the SG^−/−^ M‐BMMCs (Figure 3C). TEM ultrastructural analysis, as expected, demonstrated fewer electron‐dense MC granules in uninfected SG^−/−^ M‐BMMCs (Figure 3D). For both infected WT and SG^−/−^ M‐BMMCs, *S*.Tm could be found within visible membrane vacuoles (arrows pointing upwards), as well as seemingly free in the cytosol (arrows pointing downwards) (Figure 3E). Finally, multiple bacteria inside or in close contact with MC granules could be detected (Figure 3E). Taken together, our comparisons of infections in WT and SG^−/−^ M‐BMMCs show that MC granule dysfunction causes a markedly higher susceptibility to *S*.Tm colonization of both vacuolar and cytosolic intracellular niches.

The large differences in intracellular *S*.Tm burden between otherwise isogenic WT and serglycin‐deficient M‐BMMCs allowed us to revisit the impact of intracellular life cycle events on the MC cytokine output. Remarkably, the IL‐6 cytokine response to *S*.Tm^*wt*^ appeared essentially identical between WT and SG^−/−^ M‐BMMCs, as assayed both at 4 and 24 h p.i. (Figures 3F,G). No significant response to *S*.Tm^*∆invG*^ was noted for either genotype (Figure 3F,G; in line with Figure 1K,L). Hence, (1) the dense granulation of murine mucosal MCs restricts *S*.Tm colonization of both vacuolar and cytosolic compartments, (2) but such events are fully uncoupled from the magnitude of the MC cytokine response elicited by *S*.Tm invasion.

### 2.4 Human LUVA Cells Respond to *Salmonella* Typhimurium by Combining TLR2/6 and TTSS‐1‐Driven Signals

**FIGURE 4:**
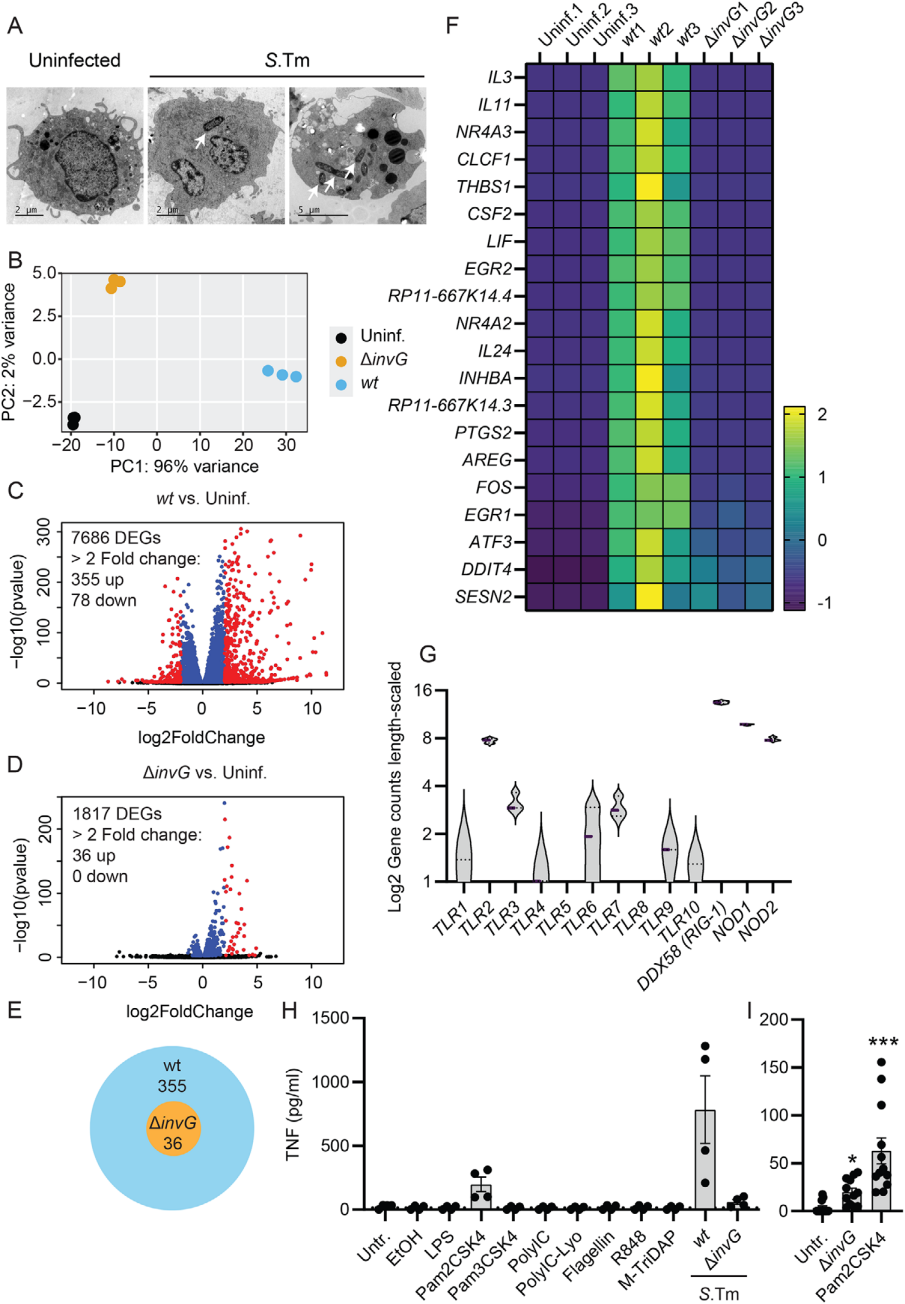
Human LUVA mast cells respond to *S*.Tm by a two‐step activation mechanism mediated by TLR2/6 and the TTSS‐1 invasion machinery. (**A**) Representative (from two experiments) TEM images of LUVA cells uninfected or infected for 4 h with *S*.Tm (white arrows) at MOI 50. (**B**) Principal component analysis (PCA) plot of RNA sequencing data, showing the uninfected group as well as 4 h p.i. with *S*.Tm^*wt*^ or *S*.Tm^*∆invG*^ (*n* = 3, from one experiment. Same for (**C–G**). (**C, D**) Volcano plots showing the number of differentially expressed genes (DEGs, p < 0.01) with > two‐fold (log2) changed genes in red and their respective number of up‐ and downregulated genes. (**C**) shows *S*.Tm^*wt*^ vs. uninfected, D shows *S*.Tm^*∆invG*^ vs. uninfected. (**E**) Venn‐diagram‐like plot showing the (complete) overlap of > two‐fold (log2) upregulated DEGs. (**F**) Heatmap of top 20 upregulated genes with baseMean > 50 and *p* < 0.01 by *S*.Tm^*∆invG*^, sorted descending by *S*.Tm^*wt*^/*S*.Tm^*∆invG*^ ratio (z‐score transformed). (**G**) Violine plots of length‐scaled gene expression values of different PRR receptors in uninfected LUVA MCs. (**H**) Levels of secreted TNF from LUVA MCs either untreated or treated for 4 h with 0.5 µg/mL LPS, 10 µg/mL Pam3CSK4 or its vehicle (0.5% ethanol), 0.1 µg/mL Pam2CSK4, 5 µg/mL Poly(I:C) (HMW), 0.5 µg/ml Poly(I:C) (HMW)/LyoVec, 5 µg/mL R848, 0.1 µg/mL *S*.Tm flagellin or 5 µg/ml M‐TriDAP as well as LUVA MCs infected with MIO 50 *S*.Tm*^wt^* or *S*.Tm^*∆invG*^, (*n* = 4 from two experiments). (**I**) Separate experiment with a similar setup as (**H**), including the untr., Pam2CSK4 and *S*.Tm^*∆invG*^ groups (*n* = 12, from three experiments). (**H**) Graph shows mean ± SEM for pooled replicates from two experiments (*n* = 4). Untreated cells were compared with other groups by one‐way ANOVA and Dunnett's post hoc test. (**I**) The Graph shows mean ± SEM for pooled replicates from three experiments (*n* = 12). Untreated cells were compared with other groups by the Kruskal–Wallis test. *p < 0.05, ***p < 0.001; ns, nonsignificant.

Lastly, we adopted LUVA cells as a model for human MC biology [27]. We previously established that LUVA cells respond to invasive and noninvasive *S*.Tm with differential levels of TNF secretion (strong vs. weak), similarly to the cytokine response observed in mouse MCs [11], and in line with our two‐step activation model. LUVA MCs are overall weakly granulated with only a few granules in a subset of cells, and can be invaded by *S*.Tm (Figure 4A, [11]). To further probe the basis for the MC cytokine response in LUVA cells, we performed RNA sequencing following 4 h of infection with *S*.Tm^*wt*^ or *S*.Tm^*∆invG*^, as was done in our previous study on murine IL3‐BMMCs [11]. A principal component analysis plot based on all differentially expressed genes (DEGs) revealed distinct clustering of the experimental groups, with *S*.Tm^*wt*^‐infected samples differing to a higher degree, and *S*.Tm^*∆invG*^‐infected samples to a lower degree, from the uninfected LUVA MC controls (Figure 4B). This was also reflected in the significantly upregulated genes (*p* < 0.01) with a base mean >50 to exclude genes with minimal expression. While *S*.Tm^wt^ induced 7686 DEGs, with 355 up‐ and 78 downregulated genes (|fold change| >2) (Figure 4C), *S*.Tm^*∆invG*^ induced 1817 DEGs with 36 up‐ and 0 downregulated genes (Figure 4D). When comparing the DEGs of both conditions, we observed no genes solely induced by *S*.Tm^*∆invG*^ (Figure 4E). Next, we examined the transcriptional activation pattern of LUVA MCs based on the 20 highest DEGs induced by *S*.Tm^*∆invG*^ (Figure 4F), or a selection of upregulated cytokines (Figure S7A). From this, it became further evident that a response to extracellular *S*.Tm^*∆invG*^ was detectable, but relatively weak compared with the full‐blown response to invasive *S*.Tm^*wt*^ (Figure 4F). Overall, the global transcriptome analysis indicates a broadly similar pattern of two‐step MC activation in LUVA cells as observed in the murine CT‐BMMC and IL‐3‐BMMC models (Figure 1, [11]).

To identify the PRR(s) responsible for the LUVA cell cytokine response to noninvasive *S*.Tm^*∆invG*^, we first examined TLR4‐mediated LPS sensing, as this was shown to be relevant for the murine CT‐BMMCs (e.g., Figure 1N). Since LUVA cells exhibited no TNF secretion when treated with pure LPS (Figure S7B), we preincubated the cells for 1 h with human recombinant CD14, which has been shown to be necessary for LPS sensing in some human MC contexts [28]. In addition, we cultured the LUVA cells for 1 additional week with IL‐4 to induce a maximally mature phenotype [27]. However, under no conditions did we observe above‐background TNF secretion upon treatment with LPS (Figure S7B). From mining of the RNA sequencing data, LUVA MCs were found to express detectable transcript levels for all TLRs, except *TLR5* and *TLR8*, including relatively high levels of transcripts encoding *TLR2* as well as the intracellular PRRs RIG‐I, NOD1, and NOD2 (Figure 4G). To screen for TNF secretion in a similar range as observed upon *S*.Tm^*∆invG*^ exposure, we therefore treated LUVA MCs with ligands for all these TLRs/PRRs. Most of the ligands elicited no detectable TNF secretion (Figure 4H). Notably, whereas the TLR1/2 ligand Pam3CSK4 was among those that did not elicit any TNF cytokine secretion, the chemically related TLR2/6 ligand Pam2CSK4 triggered higher than baseline TNF levels (Figure 4H). Furthermore, in a side‐by‐side comparison with increased sample size and a statistical analysis based on differences from untreated cells, 100 ng/mL of Pam2CSK4 induced a TNF secretion response in a similar range (∼twofold higher) as *S*.Tm^*∆invG*^ (Figure 4I). From these observations, we postulate that human LUVA MCs, in analogy to murine IL‐3‐ and CT‐BMMCs, can respond to either noninvasive and invasive *S*.Tm by differential cytokine production, but rely on a distinct TLR signaling module that includes TLR2/6.

## 3 Discussion

MCs reside in both mucosal and submucosal locations of the gut wall at steady state [4], and encounter tissue‐invading *S*.Tm following oral infection [11, 12]. By integrating TLR sensing with signals elicited downstream of bacterial TTSS‐1 effectors, MCs can tune their inflammatory cytokine output to the severity of the bacterial insult, mustering a modest response to extracellular enterobacteria and a strong response to host‐cell‐invading *S*.Tm [11]. However, MC phenotypes and gene expression patterns are heterogenous within adult tissues, with poorly understood implications for infection responses. Recent single‐cell RNA sequencing and lineage tracing have substantiated the presence of two major MC subtypes in mice [4, 29]. M‐MCs reside in superficial layers of mucosae, express high levels of the proteases Mcpt1 and Mcpt2, and are replenished from bone‐marrow progenitors in the adult [4, 29, 30]. By contrast, CT‐MCs can be found in deeper layers of the gut wall (as well as in peritoneum, lung, skin, and other connective tissues), are marked by the Mrgprb2 receptor, and originate predominantly from embryogenesis [4, 29]. Human MC heterogeneity falls more along a spectrum, with at least six clusters identified in single‐cell RNA sequencing datasets [4, 5]. In this work, we have explored how variability in mammalian MC subtype features, for example, differential PRR expression and a varying degree of granulation, impacts the interplay with the prototype enterobacterium *S*.Tm. Our study offers the following insights: (1) Diverse mouse MC subtype models and the human LUVA MC line all respond to host‐cell‐invading *S*.Tm by producing inflammatory cytokines such as IL‐6, IL‐13, and/or TNF; (2) MCs recognize extracellular enterobacterial PAMPs in an MC‐subtype‐specific manner due to differential TLR expression; (3) cultured murine M‐MCs uniquely fail to produce a cytokine response to extracellular *S*.Tm, explained by minimal TLR2 and TLR4 expression compared with the corresponding CT‐MCs; (4) *S*.Tm establishes an intracellular foothold in both vacuolar (major) and cytosolic (minor population) compartments of MCs, with M‐MCs less permissive to the bacterial colonization than CT‐MCs; (5) granular integrity maintained by the proteoglycan core protein serglycin limits *S*.Tm escape into and/or colonization of the MC cytosol across MC subtypes; and (6) the magnitude of the inflammatory MC cytokine response, represented by IL‐6 in mouse MCs or TNF in human LUVA cells, can be uncoupled from such intracellular *S*.Tm infection cycles events. These findings are further contextualized below.

As a starting point, we adapted differential murine BMMC culture protocols [19, 20, 21, 22, 23]. The IL‐3‐, CT‐, and M‐BMMC models generated in this way displayed expression of the relevant protease and receptor transcripts (e.g., Mcpt1 and Mcpt2 for M‐BMMCs and Mcpt5 and Mrgprb2 for CT‐BMMCs) and exhibited granulation clearly visible by TEM. M‐BMMCs appeared most mature in terms of both protease content, granule numbers, and granule density. Inducing an optimal in vivo‐like CT‐MC phenotype proved more challenging, with, for example, *Mcpt4* expression remaining low. This might be explained by that in vivo, murine MC progenitors in bone marrow have an inherent propensity to differentiate toward M‐MCs, while CT‐MCs instead arise from tissue‐resident progenitors of embryonic origin [4]. Other studies have used similar protocols [19, 20, 21, 22, 23], yet limited data on the resulting protease expression are available in the literature for comparison. We, moreover, established that 10 ng/mL of IL‐4 markedly improved murine CT‐BMMC maturation over the 1–2 ng/mL concentration commonly used in earlier studies [22, 24].

When assessing the early cytokine response to *S*.Tm infection, notable differences were observed between murine M‐BMMCs and CT‐BMMCs. While both subtypes responded to *S*.Tm^*wt*^ infection by cytokine (IL‐6) secretion, this response was ∼20‐fold more potent for CT‐BMMCs. Even more strikingly, murine M‐BMMCs remained completely unresponsive to noninvasive *S*.Tm^*∆invG*^, whereas CT‐BMMCs (and also IL‐3‐maintained BMMCs) produced intermediate cytokine levels upon detecting this strictly extracellular strain. This dichotomy between MC subtypes could be resolved by that CT‐BMMCs express far greater levels of *Tlr4* and *Tlr2* transcripts, and were found to respond efficiently to the cognate TLR agonists LPS and Pam3CSK4, which was not the case for M‐BMMCs. It should be mentioned here that Benedé et al. [22] cultured murine M‐BMMCs using a slightly altered version of this protocol and found them responsive to LPS stimulation under exogenous cytokine‐free conditions. We reason that subtle differences at either the start (i.e., time until adding the MC subtype differentiation‐promoting factors) or the end (i.e., the continued presence of those factors) of the culture protocol may result in an intermediate phenotype between the CT‐BMMC and M‐BMMC extremes. For example, TGF‐β has been shown to suppress IL‐33 and IgE‐mediated activation of MCs [31, 32]. Hence, sustained TGF‐β signals, as included throughout in our M‐BMMC culture regimen, could be a requirement to sustain M‐BMMC inertness also against enterobacterial PAMPs. This explanation gains support from our M‐BMMC phenotype reversal experiments, where both *Tlr4* expression and responsiveness to *S*.Tm*^∆^* ^*invG*^ and pure LPS reemerged following IL‐9 and TGF‐β withdrawal from the M‐BMMC medium. Nevertheless, we conclude that variability in TLR expression can shift murine MCs between phenotypic states responsive either solely to invasive *S*.Tm (as noted here for M‐BMMCs), or to both invasive and noninvasive *S*.Tm (as noted here for IL‐3‐BMMCs, CT‐BMMCs, and CT‐PCMCs) (current study, [11]). Furthermore, our experiments in human LUVA MCs demonstrate that the basic principle underlying two‐step MC activation—that is, integrating TLR recognition of any *S*.Tm plus sensing the TTSS‐1 activities of invading *S*.Tm—is conserved between mouse and human, but that the exact TLRs involved can differ between species or MC subtypes.

An intriguing remaining issue is to what extent the phenotypes of the MC models studied here track those of the MC subtypes found in vivo. However, data comparing the gene expression profiles of CT‐MCs and M‐MCs in vivo is still scarce. In one study, though, differences in mRNA expression between intraepithelial and submucosal MC populations during nematode infection were studied [19]. It was found that these populations display mixed phenotypes, indicating an overlap of CT‐MCs and M‐MCs. Notably, the intraepithelial MCs displayed high transcript levels for *Mcpt1/2*, but low levels for *Tlr2/4*, which agrees well with our observations in M‐BMMCs. In addition, a recent analysis failed to detect the presence of any TLR in either human MCs or in mouse peritoneal MCs at the protein level [33]. At the same time, there is ample evidence (including the present work) that multiple MC models are in principle responsive to TLR agonists. Future work will have to firmly establish the extent to which discrete MC subpopulations found in vivo express TLRs, and to clarify how TLR expression differences translate into functional responses. Our present study provides a conceptual framework for such future exploration.

Following entry into MCs, *S*.Tm^*wt*^ were found lodged predominantly in a vacuolar compartment, but with a subpopulation escaping into the MC cytosol. This appears broadly in line with the previously reported bimodal lifestyle of *S*.Tm in epithelial cells [16]. TEM analysis revealed instances of fusion between *S*.Tm‐containing vacuoles and MC granules. In addition, our fluorescent reporter assays showed that more heavily granulated M‐BMMCs restricted intracellular *S*.Tm loads better than the less granulated IL‐3‐BMMCs and CT‐BMMCs. To gain further insight into the interactions between intracellular *S*.Tm and MC granule constituents, we turned to BMMC models deficient in all major CT‐MC proteases (4KO; [26]), or in overall granule organization (SG^−/−^; [21, 34]). While no obvious antibacterial effect could be assigned to the Mcpt4‐6 and Cpa3 proteases, it remains possible that insufficient expression in WT BMMC cultures (e.g., for Mcpt4) precluded the scoring of subtler phenotypes. Nevertheless, deletion of the serglycin, the core protein that organizes the MC granule proteoglycan scaffold, resulted in highly elevated *S*.Tm colonization of both vacuolar and cytosolic compartments, as well as an elevated frequency of MC death following infection. This demonstrates that serglycin has a protective role against MC colonization by the prototype enteropathogen *S*.Tm, although the underlying mechanism remains unclear. One possible scenario could be that the protective effect of serglycin is indirect, being mediated by any of the compounds stored in a complex with serglycin. Such compounds include, beyond proteases, also biogenic amines and iron [35]. An alternative scenario could be that serglycin impacts the bacteria directly, for example, by virtue of its remarkably high negative charge density [35]. Yet another possibility could relate to the notion that serglycin maintains the osmotic pressure of the granules [36], and that *S*.Tm colonization could benefit from defective osmotic regulation in its absence. Further investigations will be needed to fully understand the mechanism by which serglycin limits *S*.Tm colonization of MCs, and whether serglycin could mediate similar effects also in other cell types (e.g., macrophages).

Finally, the elevated frequency of vacuolar, and even more so of cytosolic, *S*.Tm in serglycin‐deficient MCs allowed us to assess if such intracellular events affected the acute MC cytokine response to the bacterium. Remarkably, infected WT and SG^−/−^ MCs produced essentially identical levels of secreted IL‐6, assayed both in murine CT‐BMMC and M‐BMMC models, and at both 4 and 24 h p.i. This demonstrates that the MC cytokine response to *S*.Tm infection is dictated by early signals downstream of TLR sensing and bacterial TTSS‐1 effectors, with minimal impact of later events in the intracellular host cell colonization cycle.

### 3.1 Data Limitations and Perspectives

This study has exploited differential MC culturing to demonstrate that MC heterogeneity profoundly impacts the response to the prototype pathogen *S*.Tm. It should, however, be noted that in vivo MC heterogeneity is only beginning to be understood, as elaborated on in the discussion. Future studies building on this one will need to dissect how discrete MC populations within intact tissue affect the stepwise progression of bacterial and other infectious diseases in vivo.

## 4 Methods

### 4.1 Mice for Mast Cell Culture

Murine BMMCs and PCMCs were generated from C57BL/6 WT mice (5–14 weeks old; male and female), bred and maintained at the National Veterinary Institute (SVA, Uppsala), Sweden. SG^−/−^ mice were initially described in [21, 34] and lack serglycin. 4KO mice lack the main CTMC proteases Mcpt4, Mcpt6, and CPA3 due to genetic ablation, as well as Mcpt5 due to posttranslational effects [26]. The experimental procedures were approved by the local animal ethics committee (Uppsala djurförsöksetiska nämnd, Dnr 5.8.18‐02871.2023). Whenever possible, remaining bones from C57BL/6 WT mice used as controls in other experiments were acquired for culturing BMMCs and PCMCs.

### 4.2 Fluorescence Microscopy of Infected Caecum

We reused OCT‐embedded caecal tissue from previously published infection experiments [11]. From OCT cryoblocks stored at −80°C, new cryosections of 20 µm were cut on a Cryostat CryoStar NX70 (Epredia) with at least 40 µm distance between sections, placed on Superfrost Plus Adhesion Microscope Slides (Thermo Fisher Scientific, #J1800AMNT) and dried >16 h. To stain for Mcpt1, slides were washed 2×5 min in PBS and blocked in 1% normal goat serum (NGS, Sigma‐Aldrich, #G9023) in PBS/0.3% Triton X‐100 for 1 h at RT. 1:200 rat anti‐mouse Mcpt1 (eBioscience, #14‐5503‐82) in PBS/1% NGS/0.1% Triton X‐100 was added overnight at 4°C. On the next day, slides were washed in PBS, PBS‐Tween 0.25% and PBS for 5 min each and stained with DAPI (Sigma‐Aldrich, #D9542, 1:400), 2 U/mL phalloidin‐AF555 (Molecular Probes, #A34055), 10 µg/mL avidin‐AF488 (Thermo Fisher Scientific, #A23170) and 1:200 Goat‐α‐Rat‐IgG‐(H+L)‐AF647 (Invitrogen, #10666503) for 2 h at room temperature (RT). After washing in PBS‐PBS‐Tween‐PBS, mounting was done with Mowiol 4–88 (Sigma‐Aldrich, #81381), and slides were dried overnight before storing at 4°C. The staining with *Salmonella* O antigen (BD Difco, #226601) was optimized to retain bacteria on the slides. Sections were rehydrated for 1 min in PBS and permeabilized with PBS/0.5% Triton for 5 min. After 30 min blocking in 10% NGS and incubation with primary antiserum in 10% NGS for 40 min, slides were washed 3 × 2 min in PBS. This was followed by staining with 1:400 DAPI, 6 U/mL phalloidin‐AF647 (Molecular Probes, #A22287) and 1:200 of either Goat‐α‐Rabbit‐IgG‐(H+L)‐Cy3 (Molecular Probes, # A10520) or Goat‐α‐Rabbit‐IgG‐(H+L)‐AF488 (Molecular Probes, #A11034). After washing 3 × 2 min in PBS, slides were mounted as described above. All primary antibodies and their dilutions are shown in Table S4. Slides were imaged using an LSM700 (Zeiss) confocal microscope equipped with a Plan‐Apochromat 20×/0.8 objective with a pinhole set to 1 AU for each wavelength, at the BioVis platform of Uppsala University. Fiji [37] was used for image processing.

### 4.3 Bone Marrow‐Derived Mast Cell, Peritoneal Cell‐Derived Mast Cell, and LUVA Cell Culture

Bone marrow from tibiae and femurae of one mouse per culture was flushed out with PBS, washed 1× at 300 × *g* for 7 min, and filtered through a 70 µm cell strainer. Cells were resuspended in 50 mL BMMC culture medium consisting of 90% DMEM (Thermo Fisher Scientific, #31966047), 10% heat‐inactivated FBS (Thermo Fisher Scientific, #11573397, #10500–064), and supplemented with Penicillin‐Streptomycin (100 U/mL, 100 µg/mL, Sigma‐Aldrich, #P0781) and 10 ng/mL recombinant IL‐3 (Peprotech, #213‐13). Medium was exchanged every 3–4 days to a fresh flask during the first 4 weeks of culture, maintaining a cell density of ∼0.5 × 10^6^ cells/mL. Afterward, the medium was exchanged every 3–7 days. BMMCs were used during 4–10 weeks of culture. Cell numbers were determined by trypan blue (Thermo Fisher Scientific, #15250‐061) exclusion and quantified by an automated cell counter (Countess II FL, Life Technologies) or a Burker chamber (VWR). BMMCs from 4KO and SG^−/−^ mice, as well as corresponding WT control BMMCs, were cultured with 15 ng/ml IL‐3. To generate BMMCs of CTMC‐like phenotype (“CT‐BMMCs”), the bone marrow culture was supplemented with 10 ng/mL IL‐3, 20 ng/mL recombinant murine SCF (Peprotech, #250‐03), and 1 or 10 ng/mL recombinant murine IL‐4 (Peprotech, #214‐14). To generate BMMCs of MMC‐like phenotype (“M‐BMMCs”), the culture was supplemented with 1 ng/mL IL‐3, 50 ng/mL SCF, 5 ng/mL recombinant murine IL‐9 (Peprotech, #219‐19), and 1 ng/mL recombinant murine TGF‐β (R&D Systems, #7666‐MB‐005) in 4 mM HCl to maintain its active form. For M‐BMMCs, cells were diluted to a density of 0.25 × 10^6^ cells/mL after exchanging medium. PCMCs were established from peritoneal lavage as described previously [11]. Cells were cultured in BMMC culture medium with 50 µM 2‐mercaptoethanol (Sigma‐Aldrich, #M6250) and 20 ng/mL SCF and IL‐3. Medium was changed every 3–4 days, and the cell density was adjusted to 0.5 × 10^6^ cells/mL. LUVA cells were cultured in StemProTM‐34 SFM (Thermo Fisher Scientific, #10639011) supplemented with 1% GlutaMax (Thermo Fisher Scientific, #35050038) and 1% Penicillin‐Streptomycin. Cells were passaged every 2–4 days. When indicated in the Supporting Information figure legend, 10 ng/mL human recombinant IL‐4 was added to the culture medium (Peprotech, #200‐04).

### 4.4 *Salmonella* Typhimurium Strains, Plasmids, and Culture Conditions

All strains and plasmids used in this study can be found in Tables S1 and S2, respectively. All strains used in this study were of an SL1344 background (SB300; streptomycin resistant) [38]. For infections, *S*.Tm cultures were grown overnight at 37°C for 12 h in LB 0.3 M NaCl with appropriate antibiotics on a rotating wheel incubator to optimize aeration, followed by subculturing in the same medium without antibiotics at a 1:20 dilution for 4 h at 37°C. Prior to infection, 1 mL was spun down for 4 min at 12,000×*g* and reconstituted in co‐cultivation medium (MC‐subtype‐specific standard culture medium without antibiotics; see above for details). After infection, inocula were diluted 1:10^6^, and 50 µL were plated onto agar plates with antibiotics when appropriate to enumerate colony‐forming units (CFU).

### 4.5 Infection of Mast Cells with *Salmonella* Typhimurium

For all infections, MCs were washed twice in PBS and resuspended in the respective culture medium without antibiotics (co‐cultivation medium). In experiments performed for ELISA or RT‐qPCR analysis, if not indicated otherwise, 500 µL of 1 × 10^6^ MCs/mL were added to 24‐well plates (Sarstedt) and infected with 25 µL inoculum, resulting in a multiplicity of infection (MOI) of 50, for 30 min at 37°C, 5% CO_2_. Afterward, 25 µL gentamicin was added to a final concentration of 90 µg/mL, leaving the intracellular bacteria unaffected. After an additional 3.5 h, wells were harvested in tubes, centrifuged for 5 min at 400×*g*, and supernatants and pellets were frozen separately. To generate samples for immunoblots, pellets were washed with PBS once before freezing. For experiments involving flow cytometry, 180 µL of 0.556 × 10^6^ MCs/mL (for a final concentration of 0.5 × 10^6^ MCs/mL in 200 µL) were added to 96‐well round‐bottom plates (Thermo Fisher Scientific, #163320) and infected by adding 20 µL of bacteria to the indicated MOIs. After 30 min of incubation as above, plates were gently centrifuged for 3 min, 200 x g, supernatants were discarded, and the plates were vortexed gently before adding 200 µL/well of co‐cultivation medium, containing 100 µg/mL gentamicin. Plates were incubated for a further 3.5 h, washed 1× in 1% BSA (Sigma‐Aldrich # A9418) in PBS (200×*g*, 3 min), and fixed in 2% PFA (Sigma‐Aldrich, #158127) in the dark for 20–30 min. After 1× washing, cells were resuspended in 1% BSA in PBS and stored at 4°C until flow cytometry analysis of GFP‐positive MCs. The PRR agonists *E. coli* LPS (TLR4, Sigma‐Aldrich, #L4516), Pam2CSK4 (TLR2/6, Tocris, #4637), Pam3CSK4 (TLR1/2, Tocris, #4633), Poly(I:C) HMW (TLR3, InvivoGen, #tlrl‐pic), Poly(I:C) (HMW)/LyoVec (RIG‐I, InvivoGen, #tlrl‐piclv), S.*Tm* flagellin (FliC, TLR5, Sigma‐Aldrich, #SRP8029), R848 (TLR7/8, InvivoGen, #tlrl‐r848‐1), and M‐TriDAP (NOD1/2, InvivoGen, #tlrl‐mtd) were added diluted in medium and used as indicated in the respective figure legend. When indicated in the figure, human recombinant soluble CD14 (Peprotech, #110‐01) was added to the culture medium for 1 h prior to treatment with LPS. For 24 h infections of BMMCs, as well as for 4 h infections of different BMMC subtypes, the co‐cultivation medium was supplemented with cytokines at half the concentration used in culture. For infections of LUVA, a final cell density of 1.14 × 10^6^ cells/mL was used in 12‐ or 24‐well plates to mirror the same cell‐to‐surface ratio as in the 96‐well setup. ELISAs were from Invitrogen (mouse TNF #88‐7324‐88, IL‐6 #88‐7064‐88, IL‐13 #88‐7137‐88, Mcpt1 #88‐7503‐88 and human TNF #88‐7346‐88).

### 4.6 Transmission Electron Microscopy

For TEM analysis, 1 × 10^6^ BMMCs or LUVA infected with *S*.Tm MOI 50 for 4 h (gentamicin present after 30 min), or left uninfected, were washed once in 1% BSA in PBS and fixed in 2.5% Glutaraldehyde (Ted Pella) + 1 % Paraformaldehyde (Merck) in PIPES pH 7.4 and stored at 4°C until further processed. Rinsing for 10 min with 0.1 M phosphate buffer (PB) was followed by 1 h incubation in 1% osmium tetroxide (TAAB) in 0.1 M PB. After rinsing in 0.1 M PB, samples were dehydrated using an ethanol gradient (50%, 70%, 95%, and 99.9%) for 10 min at each step, followed by 5 min propylene oxide (TAAB) incubation. Afterward, samples were placed in a mixture of Epon Resin (Ted Pella) and propylene oxide (1:1) for 1 h, followed by 100% resin incubation overnight. Subsequently, samples were embedded in capsules in newly prepared Epon resin and incubated for 1–2 h, and polymerized at 60°C for 48 h. Ultrathin sections (60–70 nm) were cut in an EM UC7 Ultramicrotome (Leica) and placed on a grid. 5% uranyl acetate and Reynold's lead citrate were used for contrasting and visualized with Tecnai G2 Spirit BioTwin transmission electron microscope (Thermo Fisher/FEI) at 80 kV with an ORIUS SC200 CCD camera and Gatan Digital Micrograph software (both from Gatan Inc.).

### 4.7 Real‐Time Quantitative PCR

Total RNA was isolated from frozen pellets using the RNeasy Plus Mini Kit (Qiagen, #74134), followed by on‐column DNA digestion by the RNase‐Free DNase Set (Qiagen, #79254). The RNA concentration was measured by NanoDrop. RNA up to 1 µg was reverse transcribed into cDNA using the iScript cDNA Synthesis Kit (Bio‐Rad, #1708891) and stored at −20°C. cDNA was diluted 1:5, and qPCR was performed on a CFX384 Touch (Bio‐Rad) with iTaq Universal SYBR (Bio‐Rad, #1725121) using 1 µL of cDNA and 200 nM of reverse and forward primers (primer sequences listed in Table S3). PCR was performed according to the manufacturer's instructions. *Gapdh* was used as a housekeeping gene, and statistics were calculated based on ΔCq values.

### 4.8 Flow Cytometry

For cell surface staining of FcεRI, CD117, or CD63, 1 × 10^6^ BMMCs were resuspended in 1% BSA in PBS, containing 0.5 µg/mL of Fc‐blocking anti‐CD16/32 (1:1000, BD Life Sciences, #553142) and blocked for 5 min at RT. MCs were either left unstained or stained with anti‐FcεR1 alpha PE‐Cyanine7 (1:200, Invitrogen, #25‐5898‐82) or anti‐CD117 BV421 (1:200, BD, #566290). After 30 min incubation on ice, the cells were washed and resuspended in 1% BSA in PBS. For Mcpt1 staining, cells were fixed for 20 min in 4% PFA in the dark and afterwards resuspended in 0.1% saponin (VWR, Calbiochem, #558255), 1% BSA, and anti‐Mcpt1 (1:200, eBioscience, #14‐5503‐82) for 1 h at RT in the dark. After washing in 0.1% saponin, 1% BSA in PBS, the MCs were incubated for 30 min with 1:200 Goat‐anti‐rat‐IgG(H+L)‐Cy3 (Thermo Fisher Scientific, #A10522) in the dark and washed in 0.1% saponin again. Flow cytometry was performed using a MACSQuant VYB (Miltenyi Biotec). Data analysis was performed using FlowJo (BD Biosciences) version 10.8.1. For cell death staining, cells were resuspended in 1% BSA in PBS containing either 1:200 or 1:500 DRAQ7 (Biostatus, #DR70250) or 1:500 propidium iodide (PI) (Sigma‐Aldrich, #81845‐25MG). Antibodies used in this study are listed in Table S4. Prior to the gating shown in the figures, single cells were gated based on FSC‐A and FSC‐H.

### 4.9 Protein Extraction and Immunoblotting

Frozen pellets were resuspended in cold lysis buffer consisting of RIPA buffer (Thermo Fisher Scientific, #89900) with protease inhibitors (Roche, #4693132001), using 75 µL per 1 × 10^6^ cells. After 30 min incubation on ice, samples were centrifuged for 20 min at 4°C, maximum speed on a tabletop centrifuge, to remove debris. Supernatants were transferred to fresh tubes, protein concentration measured with the bicinchoninic acid assay (Pierce, #23227), and the samples were either directly processed or frozen until further use. RIPA lysates were mixed with 4× Laemmli buffer (Bio‐Rad, #1610747) containing 5% 2‐mercaptoethanol, boiled for 5 min at 95°C, and loaded together with 5 µL of ladder (Bio‐Rad, #1610373) on 4%–20% Mini‐PROTEAN TGX Stain‐Free Protein Gels (Bio‐Rad, #4568094 or #4568096). Gels were run for 35 min at 200 V and transferred to nitrocellulose (Bio‐Rad, #1704158) with a Trans‐Blot Turbo Transfer System (Bio‐Rad), using the program for intermediate molecular weight. One‐minute activation of the gel and imaging of total protein on the blot were performed by a Gel Doc EZ Imager (Bio‐Rad). Blots were blocked in Intercept blocking buffer (PBS, LI‐COR, #927‐70001) for 1 h at RT, incubated 2 h on RT or overnight at 4°C with in‐house rabbit anti‐sera (polyclonal) for Mcpt2 or Mcpt6 (1:1000) diluted in blocking buffer. After 3× washing in PBS‐Tween (0.05%), blots were incubated with IRDye 800CW Donkey anti‐Rabbit IgG (1:10,000, LI‐COR, #926‐32213) for 1 h at RT in the dark. After washing, blots were imaged with the Odyssey CLX imaging system (LI‐COR), and the signal was normalized to total protein on the membrane or used as a control for the amount of protein loaded.

### 4.10 RNA Sequencing

Sequencing libraries were prepared from 500 ng total MC RNA using the TruSeq stranded mRNA library preparation kit (Illumina Inc., #20020595), including polyA selection. Unique dual indexes (Illumina Inc., 20040871) were used, and the library preparation was performed according to the manufacturer's protocol (#1000000040498). The quality of the libraries was evaluated using the Fragment Analyzer (Advanced Analytical Technologies Inc.) and the DNF‐910 dsDNA kit. The adapter‐ligated fragments were quantified by qPCR using the library quantification kit for Illumina (KAPA Biosystems) on a CFX384 Touch instrument (Bio‐Rad) prior to cluster generation and sequencing. Library preparation and sequencing were performed by the SNP&SEQ Technology Platform, a national unit within the National Genomics Infrastructure (NGI), hosted by Science for Life Laboratory, in Uppsala, Sweden (scilifelab.se/units/ngiuppsala). Sequencing was carried out with Paired‐end 150 bp read length on a NovaSeq X Plus system, 10B flow cell, and XLEAP‐SBS sequencing chemistry according to the manufacturer's instructions. Additional statistics on sequencing quality were compiled with an in‐house script from the FASTQ files, RTA, and BCL2FASTQ2 output files. The RNA‐seq data were analyzed using the best practice pipeline nf‐core/rnaseq. Detailed information about the analysis pipeline can be found here https://nf‐co.re/rnaseq/3.17.0/ and here nf‐co.re/sarek. For differential expression analysis, DESeq2 1.44.0 in combination with R 4.4.1 and RStudio 2024.04.2 were used. Transcriptome data can be accessed at Gene Expression Omnibus under accession number GSE285817.

### 4.11 Statistical Analysis

If not indicated otherwise, all graphs were plotted with Prism 10.2.3 (GraphPad) and statistical analysis performed as stated in the figure legends, either with one‐way analysis of variance (ANOVA) or two‐way ANOVA with Dunnett's or Sidak's post hoc tests. Significance levels were *p < 0.05, **p < 0.01, and ***p < 0.001. If not indicated otherwise, for every *n*, the mean of all MC wells infected with an individual bacterial subculture derived from an individual overnight culture serves as a single data point. If not indicated otherwise, every experiment was performed at least twice on different days.

## Author Contributions

**Christopher von Beek**: Conceptualization, methodology, investigation, formal analysis, interpretation, visualization, writing – original draft. **Grisna I. Prensa**: Methodology, investigation, formal analysis, interpretation. **Julia H. M. Andersson**: Methodology, investigation, formal analysis, interpretation. **Gunnar Pejler**: Conceptualization, interpretation, resources, supervision, funding acquisition. **Mikael E. Sellin**: Conceptualization, interpretation, resources, supervision, funding acquisition, writing – original draft. All authors scrutinized and approved the final manuscript.

## Conflicts of Interest

The authors declare no conflicts of interest.

## Peer Review

The peer review history for this article is available at https://publons.com/publon/10.1002/eji.70040.

## Supporting information




**Supporting File 1**: eji70040‐sup‐0001‐SuppMat.pdf.

**Supporting File 2**: eji70040‐sup‐0002‐SuppMat.pdf.

## Data Availability

RNA sequencing raw data that support these findings were deposited in GEO, accessible under accession number GSE285817. Immunoblot raw data are provided as a source data file. Raw data underlying all other plots presented in this study are available from the corresponding author upon reasonable request.
