# Supplementary material for: Mast Cell Phenotypic Heterogeneity Impacts the Interplay with Pathogenic Salmonella Typhimurium Bacteria
Source: Eur J Immunol. 2025 Aug 21;55(8):e70040. doi: 10.1002/eji.70040 (PMC12369454; doi:10.1002/eji.70040)
Supplement: Supplementary file 1 — Supporting File 1: eji70040‐sup‐0001‐SuppMat.pdf. [file EJI-55-e70040-s001.pdf]

Supplementary information for

"Mast Cell Phenotypic Heterogeneity Impacts the Interplay with Pathogenic *Salmonella* Typhimurium Bacteria"

Christopher von Beek[1], Grisna I. Prensa[1], Julia H. M. Andersson[1,2], Gunnar Pejler[1], Mikael E. Sellin[1,3,#]

Affiliations

[1]Department of Medical Biochemistry and Microbiology, Uppsala University, Uppsala, Sweden.

[2]Present address: Swedish University of Agricultural Sciences, Department of Animal Biosciences, Section for Parasitology, Uppsala, Sweden.

[3]Science for Life Laboratory, Uppsala, Sweden.

[#]Correspondence: M.E.S.; Mikael.sellin@imbim.uu.se

Figure S1

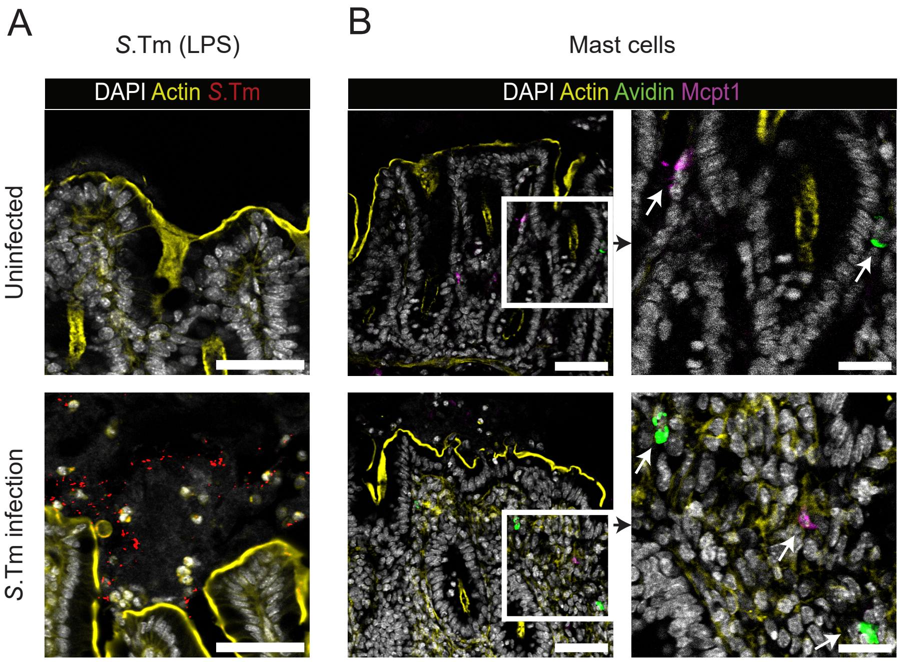

A
S.Tm (LPS)
DAPI Actin S.Tm
Uninfected
S.Tm infection
B
Mast cells
DAPI Actin Avidin Mcpt1

Figure S1. Mucosal and connective tissue mast cells are both present in uninfected and *S*.Tm-infected murine caecum tissue.

A: Representative immunofluorescence images of *S*.Tm-infected caecum tissue 42 days p.i. and untreated controls, stained for DAPI, actin and *S.*Tm LPS. Images in A are for illustrative purposes and uninfected and infected tissue section were stained under non-identical conditions (See methods). Scale bars: 50 µm. **B**: Representative immunofluorescence images of *S*.Tm-infected caecum tissue 42 days p.i. and untreated controls, stained for DAPI, actin, avidin and Mcpt1. Scale bars: 50 µm. Magnifications are in 100x100 µm, scale bars: 20 µm. Images are representative of n=3 mice per group.

Figure S2

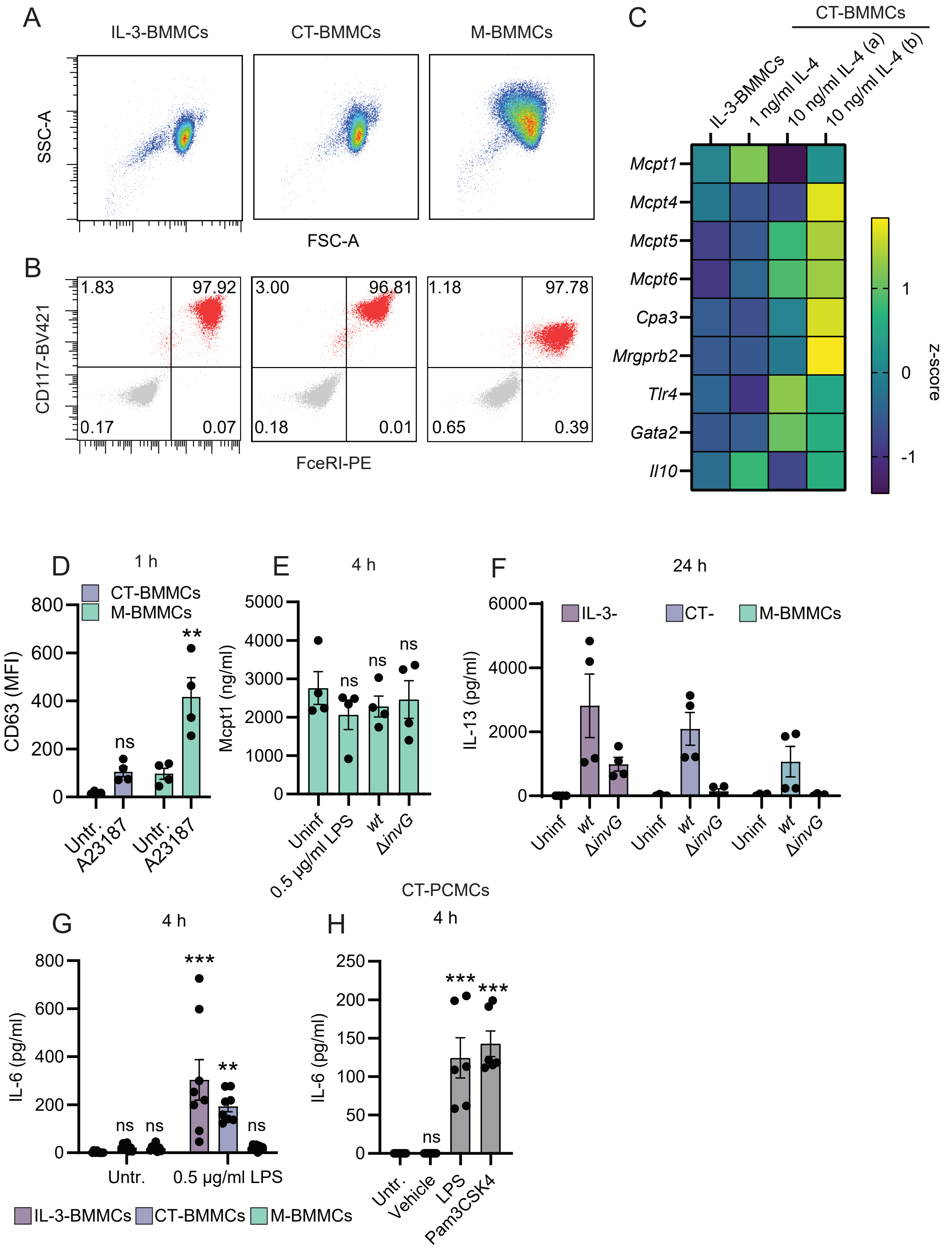
A
IL-3-BMMCs
CT-BMMCs
M-BMMCs
SSC-A
FSC-A
B
1.83
97.92
0.17
0.07
3.00
96.81
0.18
0.01
1.18
97.78
0.65
0.39
CD117-BV421
FceRI-PE
C
CT-BMMCs
IL-3-BMMCs
1 ng/ml IL-4
10 ng/ml IL-4 (a)
10 ng/ml IL-4 (b)
Mcpt1
Mcpt4
Mcpt5
Mcpt6
Cpa3
Mrgprb2
Tlr4
Gata2
Il10
1
0
-1
z-score
D
1 h
CT-BMMCs
M-BMMCs
CD63 (MFI)
ns
**
Untr.
A23187
Untr.
A23187
E
4 h
Mcpt1 (ng/ml)
ns
ns
ns
Uninf
0.5 µg/ml LPS
wt
ΔinvG
F
24 h
IL-3-
CT-
M-BMMCs
IL-13 (pg/ml)
Uninf
wt
ΔinvG
CT-PCMCs
G
4 h
IL-6 (pg/ml)
***
**
ns
ns
ns
Untr.
0.5 µg/ml LPS
IL-3-BMMCs
CT-BMMCs
M-BMMCs
H
4 h
IL-6 (pg/ml)
***
***
ns
Untr.
Vehicle
LPS
Pam3CSK4

Figure S2. Differentiation of murine bone marrow cells into bone marrow-derived mast cells with connective tissue- and mucosal-like phenotypes.

A: Representative (from 2 experiments) flow cytometry plots of forward scatter area (FSC-A) and side scatter area (SSC-A) profiles of two independent 4-week-old BMMC cultures each. **B**: Representative (from 2 experiments) flow cytometry plots of BMMC cultures stained for CD117 and FcεRI (cultures in A). **C**: Transcriptional profile of differences in BMMC phenotypes when using 1 ng/ml IL-4 in the CT-BMMC cocktail, changing to 10 ng/ml (a) or using 10 ng/ml IL-4 from the beginning of culturing (b), shown as z-scores of $2^{-\Delta Cq}$ values relative to *Gapdh* (n=4 from 2 experiments for IL-3-BMMCs, n=2 from 1 experiment for other groups). **D**: CD63 median fluorescence intensity (MFI) as proxy for baseline granulation and degranulation of CT- and M-BMMCs, untreated (Untr.) or stimulated with 1 µM A23187 for 1 h, (n=4 from 2 experiments). **E**: Mcpt1 levels of M-BMMCs treated with 0.5 µg/ml LPS, untreated (Untr.) or infected with MOI 50 of S.Tm^{wt} or *S*.Tm$^{\Delta invG}$ for 4 h, (n=4 from 2 experiments). **F**: Levels of secreted IL-13 from IL-3-BMMCs, CT-BMMCs or M-BMMCs uninfected (Uninf) or infected with MOI 50 of S.Tm^{wt} or *S*.Tm$^{\Delta invG}$ for 24 h, (n=4 from 2 experiments). **G**: Pooled responses of levels of secreted IL-6 from all LPS experiments, (n=8 from 4 experiments). Contains replotted data from Figure 1N. **H**: Levels of secreted IL-6 for CT-PCMCs untreated (Untr.)., or treated with vehicle (0.1% ethanol), 0.5 µg/ml LPS, or 10 µg/ml Pam3CSK4, (n=6 from 3 experiments). For D-G, means ± SEM are shown for pooled replicates from 2 BMMC cultures, for H, 1 CT-PCMC culture. D, F: Within each BMMC subtype, the untreated group was compared by two-way ANOVA and Sidak's posthoc test to the A23187 group. E, H: Untreated or uninfected MCs were compared by one-way ANOVA and Dunnet's posthoc test to all other groups. G: Within a MC group, Untr. groups were compared to LPS by two-way ANOVA and Tukey's posthoc test. ** $p < 0.01$; *** $p < 0.001$; ns – non-significant.

Figure S3

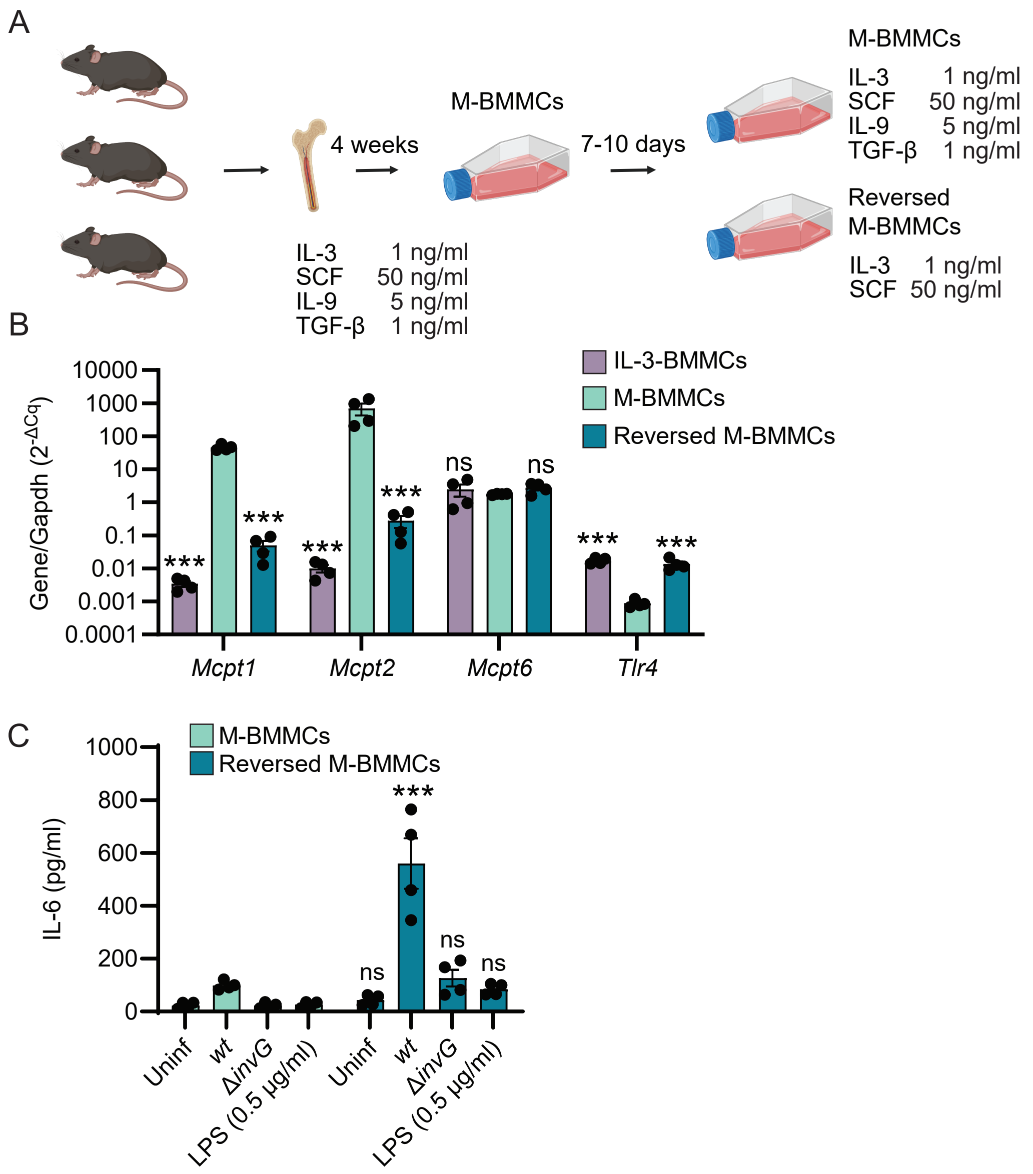
A
4 weeks
M-BMMCs
7-10 days
IL-3 1 ng/ml
SCF 50 ng/ml
IL-9 5 ng/ml
TGF-β 1 ng/ml
M-BMMCs
IL-3 1 ng/ml
SCF 50 ng/ml
IL-9 5 ng/ml
TGF-β 1 ng/ml
Reversed M-BMMCs
IL-3 1 ng/ml
SCF 50 ng/ml
B
Gene/Gapdh (2^-ΔCq)
10000
1000
100
10
1
0.1
0.01
0.001
0.0001
IL-3-BMMCs
M-BMMCs
Reversed M-BMMCs
***
ns
Mcpt1
Mcpt2
Mcpt6
Tlr4
C
IL-6 (pg/ml)
1000
800
600
400
200
0
M-BMMCs
Reversed M-BMMCs
***
ns
Uninf
wt
ΔinvG
LPS (0.5 µg/ml)

Figure S3. Murine mucosal BMMC phenotype maintenance depends on the continuous presence of IL-9 and TGF-β.

A: Experimental setup. M-BMMCs were generated for 4 weeks from bone marrow and split into two cultures for 7 (culture 1) or 10 (culture 2) days. One culture was maintained at the same culture conditions, while the second culture was deprived of IL-9 and TGF-β, with IL-3 and SCF still present ("Reversed M-BMMCs"). **B:** Transcriptional profile of IL-3-, M- and Reversed M-BMMCs, shown as gene expression relative to *Gapdh* (n=4, from 2 experiments). **C**: Levels of secreted IL-6 from M- and Reversed M-BMMCs infected with MOI 50 of S.Tm^{wt} or *S*.Tm$^{\Delta invG}$ or treated with 0.5 µg/ml LPS for 4 h (n=4, from 2 experiments). B and C show means ± SEM for 2 pooled replicates each from 2 BMMC cultures. B: M-BMMCs were compared to other groups by two-way ANOVA with Dunnet's posthoc test. C: Within each group, both BMMC subtypes were compared to each other by two-way ANOVA and Sidak's posthoc test. *** $p < 0.001$; ns – non-significant.

Figure S4

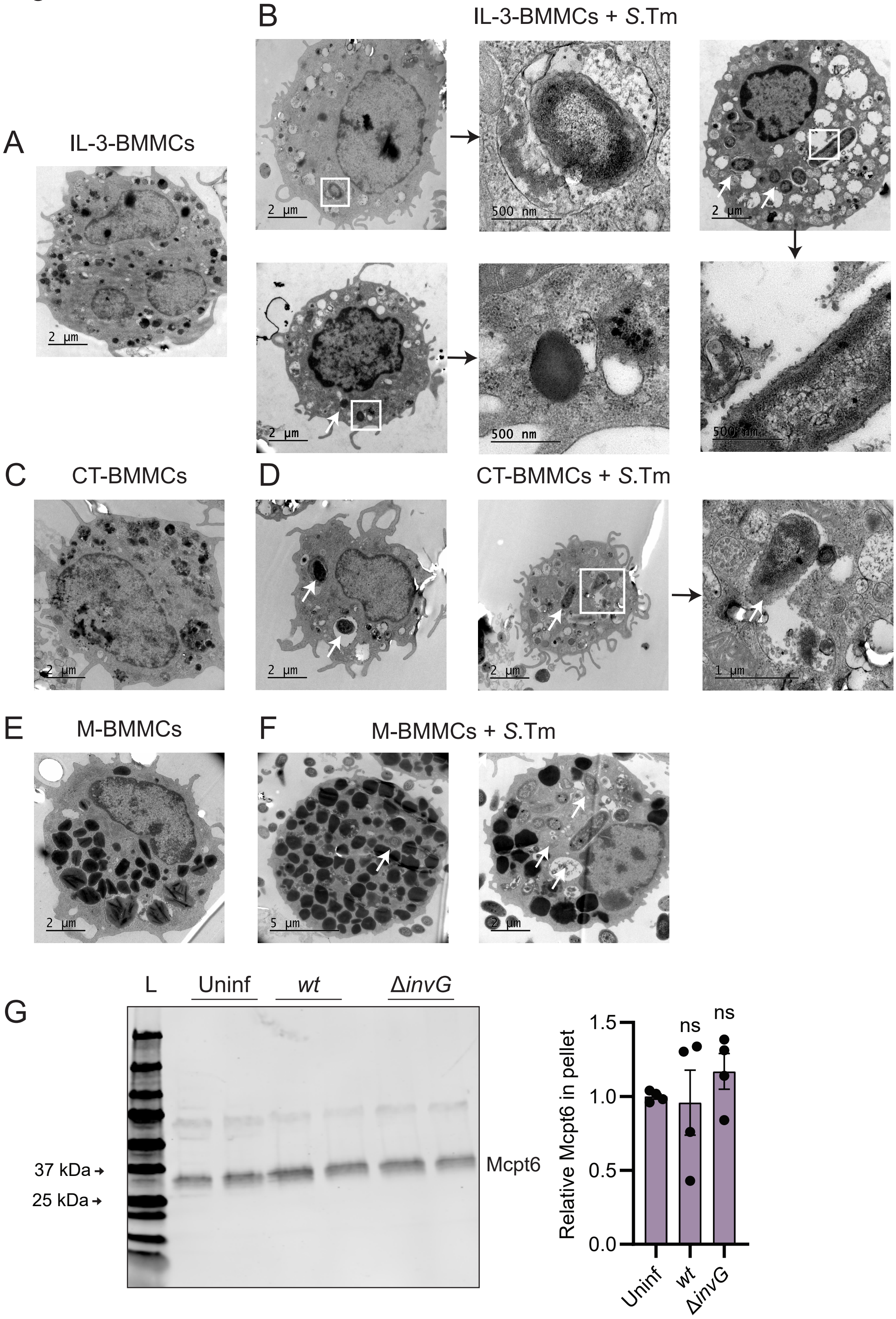

A
IL-3-BMMCs
2 μm
B
IL-3-BMMCs + S.Tm
2 μm
500 nm
2 μm
2 μm
500 nm
500 nm
C
CT-BMMCs
2 μm
D
CT-BMMCs + S.Tm
2 μm
2 μm
1 μm
E
M-BMMCs
2 μm
F
M-BMMCs + S.Tm
5 μm
2 μm
G
L
Uninf
wt
ΔinvG
37 kDa
25 kDa
Mcpt6
Relative Mcpt6 in pellet
1.5
1.0
0.5
0.0
ns
ns
Uninf
wt
ΔinvG

Figure S4. *S*.Tm invade all mast cell subtypes and *S*.Tm-containing vacuoles may fuse with granules. A, C, E: Representative (from 2 experiments) TEM images of uninfected IL-3-, CT- and M-BMMCs. **B, D, F**: Selected intracellular *S*.Tm within infected BMMC subtypes marked by arrows. Enlargements (separate TEM images of the same area in higher magnification) of regions at which bacteria-containing vacuoles fuse with MC granules (for B and D). **G**: Immunoblot analysis for Mcpt6 (left) and densitometry quantification (right) of IL-3-BMMCs 4 h p.i. with *S*.Tm^{wt} or *S*.Tm$^{\Delta invG}$ (n=4, from 2 experiments). Signal was normalized to Stain-Free total protein (see immunoblot source data). The Graph shows means ± SEM for 4 pooled replicates from 2 BMMC cultures. Uninfected cells were used for statistical comparisons by one-way ANOVA and Dunnet's posthoc test to all other groups. ns – non-significant.

Figure S5

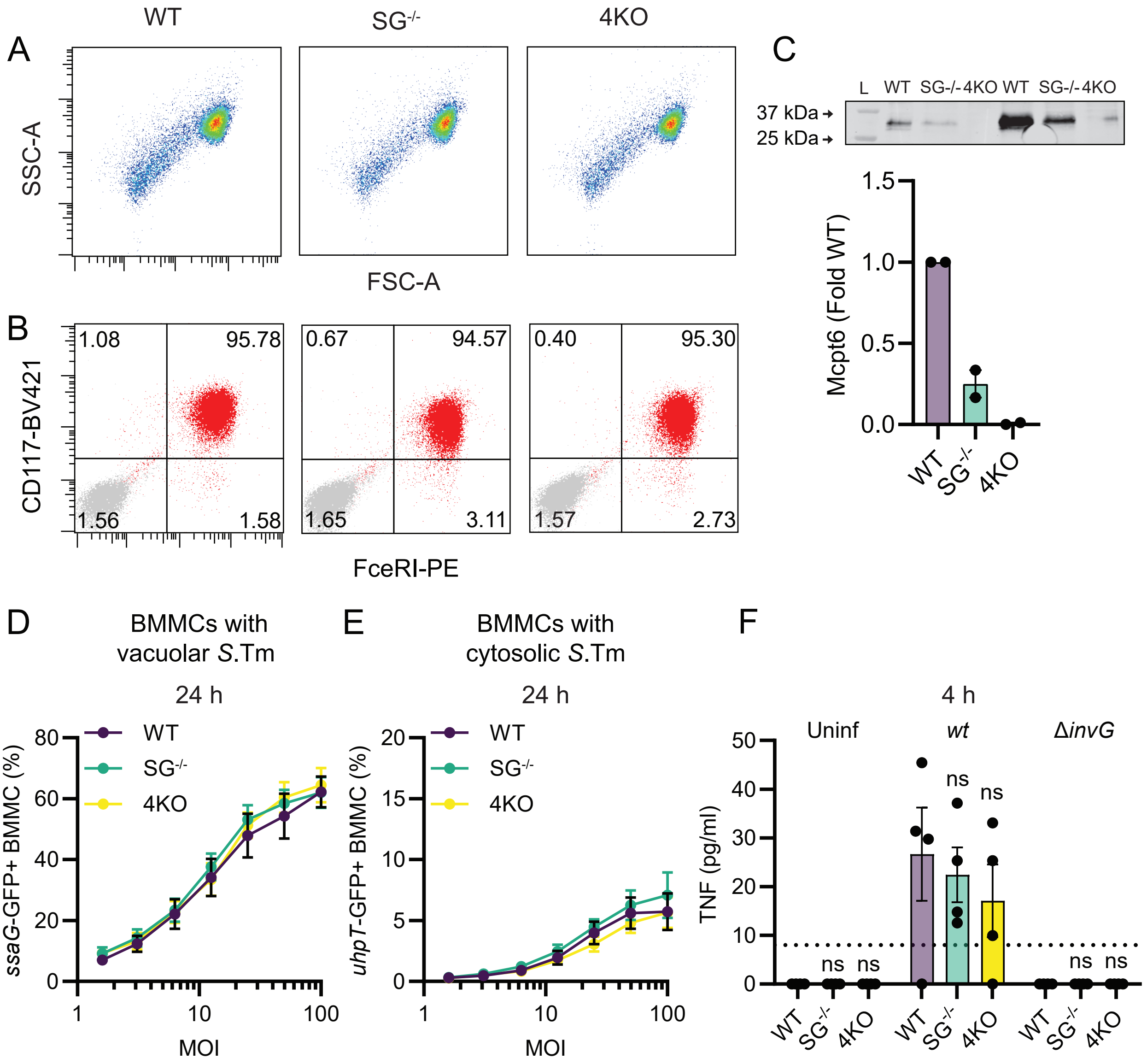

A
WT
SG-/-
4KO
SSC-A
FSC-A
B
1.08
95.78
1.56
1.58
0.67
94.57
1.65
3.11
0.40
95.30
1.57
2.73
CD117-BV421
FceRI-PE
C
L WT SG-/- 4KO WT SG-/- 4KO
37 kDa
25 kDa
Mcpt6 (Fold WT)
1.5
1.0
0.5
0.0
WT
SG-/-
4KO
D
BMMCs with vacuolar S.Tm
24 h
ssaG-GFP+ BMMC (%)
WT
SG-/-
4KO
MOI
E
BMMCs with cytosolic S.Tm
24 h
uhpT-GFP+ BMMC (%)
WT
SG-/-
4KO
MOI
F
4 h
Uninf
wt
ΔinvG
TNF (pg/ml)
ns
WT
SG-/-
4KO

Figure S5. Differentiation of murine bone marrow cells into bone marrow-derived mast cells from 4KO and SG$^{-/-}$ mice.

A: Representative (for 3 experiments) flow cytometry plots of forward scatter area (FSC-A) and side scatter area (SSC-A) profiles of 4-week-old IL-3-BMMC cultures of the indicated genotype. **B**: Representative (for 3 experiments) flow cytometry plots of IL-3-BMMC cultures stained for CD117 and FcεRI. **C**: Immunoblot analysis for Mcpt6 of 2 BMMC cultures (top) with densitometry quantification (bottom). Within each round of parallel cultured BMMCs, equal amount of protein was loaded (see immunoblot source data). **D**: Percentage of BMMCs harboring vacuolar (s*saG*-GFP+) *S.*Tm 24 h p.i. (n=6, from 3 experiments). **E**: Percentage of BMMCs harboring cytosolic (*uhpT*-GFP+) *S.*Tm 24 h p.i. (n=6, from 3 experiments). **F**: Levels of secreted TNF from BMMCs infected with MOI 50 of *S*.Tm^{wt} or *S*.Tm$^{\Delta invG}$ for 4 h (n=4, from 2 experiments). C-F show means ± SEM for pooled replicates from 2 (3 for A-B) BMMC cultures. F: WT BMMCs were compared to other groups by two-way ANOVA with Dunnet's posthoc test. * $p < 0.05$; ** $p < 0.01$; *** $p < 0.001$; ns – non-significant.

Figure S6

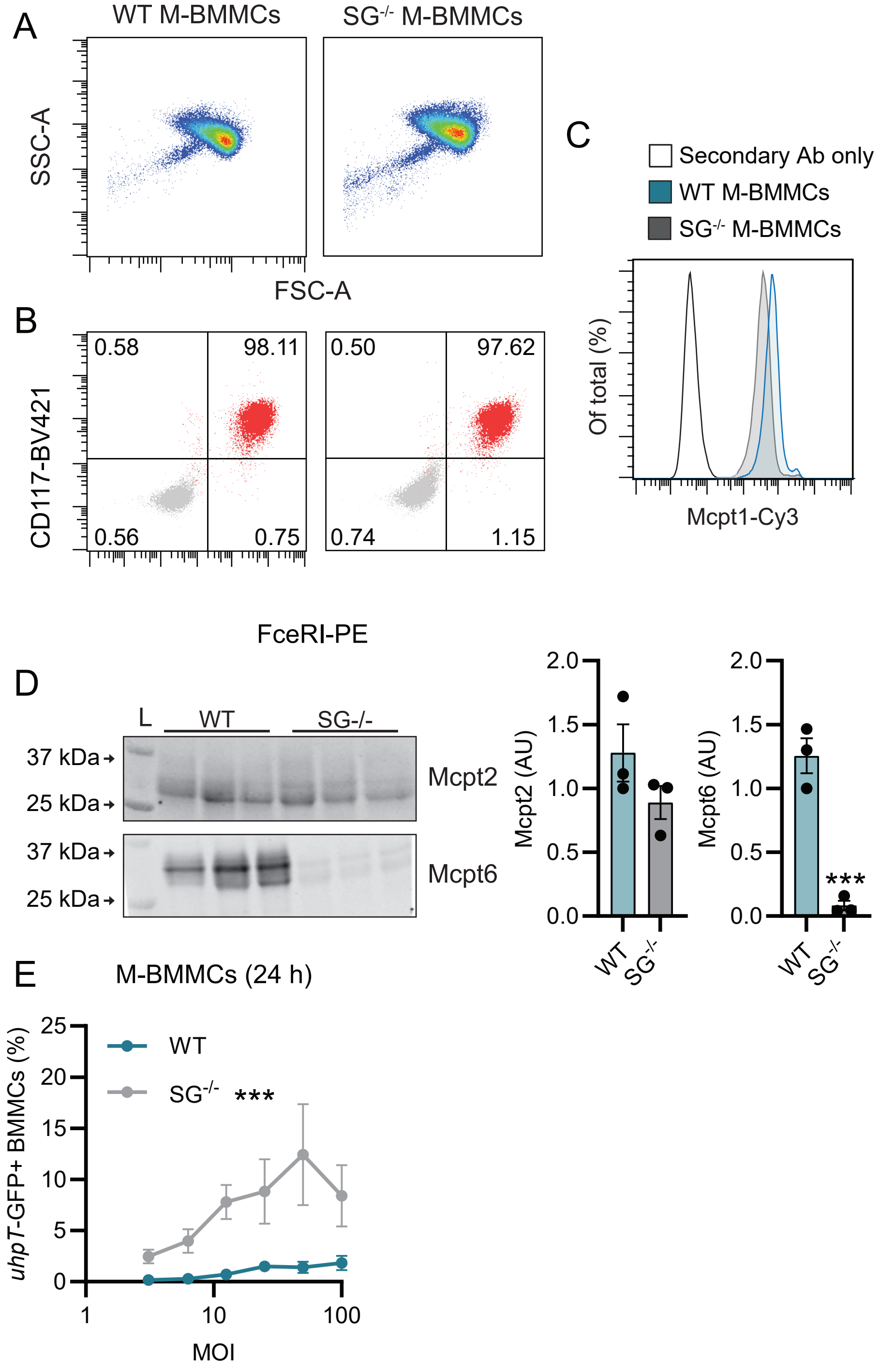
A
WT M-BMMCs
SG-/- M-BMMCs
SSC-A
FSC-A
B
0.58
98.11
0.56
0.75
0.50
97.62
0.74
1.15
CD117-BV421
FceRI-PE
C
Secondary Ab only
WT M-BMMCs
SG-/- M-BMMCs
Of total (%)
Mcpt1-Cy3
D
L
WT
SG-/-
37 kDa
25 kDa
Mcpt2
Mcpt6
Mcpt2 (AU)
Mcpt6 (AU)
2.0
1.5
1.0
0.5
0.0
***
WT
SG-/-
E
M-BMMCs (24 h)
WT
SG-/- ***
uhpT-GFP+ BMMCs (%)
25
20
15
10
5
0
1
10
100
MOI

Figure S6. Effects of serglycin deficiency in murine mucosal BMMCs on protease content and the intracellular *S.*Tm niche.

A: Flow cytometry plots of forward scatter area (FSC-A) and side scatter area (SSC-A) profiles of 4-week-old M-BMMC cultures. **B**: Flow cytometry plots of M-BMMC cultures stained for CD117 and FcεRI. **C**: Staining for intracellular Mcpt1 (same colors as in bar graphs) with white indicating secondary antibody-only control. **D**: Immunoblot for Mcpt2 and Mcpt6 (left) with densitometry quantification (right) (n=3) from 1 culture after 3-6 weeks. Protein from 200,000 cells was loaded to reflect protease content per cell. For total protein, see immunoblot source data. **E**: Percentage of M-BMMCs harboring cytosolic (*uhpT*-GFP+) *S.*Tm 24 h p.i. (n=4, from 2 experiments). D-E show means ± SEM. WT M-BMMCs were compared to SG$^{-/-}$other groups by two-way ANOVA with Dunnet's posthoc test. *** $p < 0.001$; ns – non-significant.

Figure S7

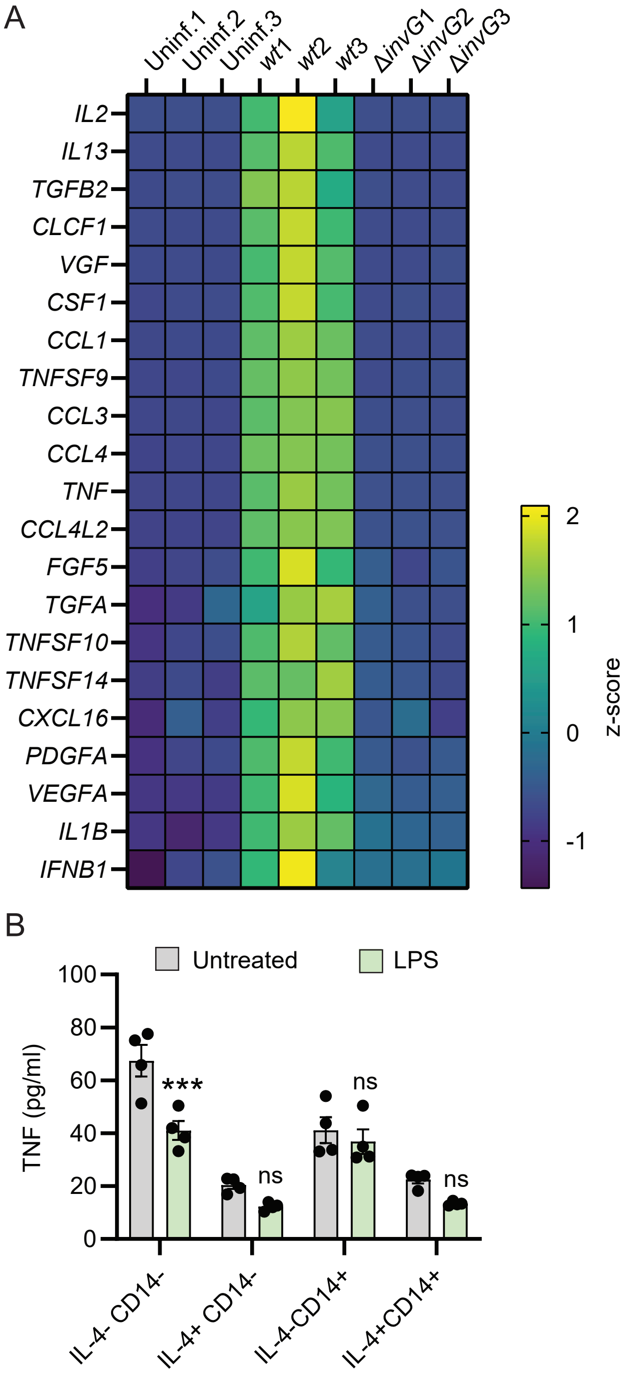
A
Uninf.1
Uninf.2
Uninf.3
wt1
wt2
wt3
ΔinvG1
ΔinvG2
ΔinvG3
IL2
IL13
TGFB2
CLCF1
VGF
CSF1
CCL1
TNFSF9
CCL3
CCL4
TNF
CCL4L2
FGF5
TGFA
TNFSF10
TNFSF14
CXCL16
PDGFA
VEGFA
IL1B
IFNB1
2
1
0
-1
z-score
B
Untreated
LPS
TNF (pg/ml)
100
80
60
40
20
0
***
ns
ns
ns
IL-4- CD14-
IL-4+ CD14-
IL-4-CD14+
IL-4+CD14+

Figure S7. Cytokines expressed by LUVA mast cells infected with *S*.Tm or after stimulation with LPS. **A:** Heatmap of 21 upregulated LUVA MC cytokine genes with baseMean > 50 and p < 0.01 by *S*.Tm^{wt}, sorted descending by *S*.Tm^{wt}/*S*.Tm$^{\Delta invG}$ ratio (z-score transformed, n=3 from 1 experiment). **B**: Levels of secreted TNF from LUVA MCs (untreated or treated with LPS), ± pretreatment for 1 h with soluble CD14 ± pretreatment for 1 week with 10 ng/ml IL-4, compared to their non-treated counterparts. The figure shows means ± SEM for pooled replicates from 2 experiments (n=4). Within each pre-treatment condition, untreated cells were compared to LPS treated cells by two-way ANOVA with Sidak's posthoc test. *** p<0.001; ns – non-significant.

Table S1. Bacterial strains and mutants used in this study. Indicated resistances as “Sm” for streptomycin. *S.*Tm^{wt} was also used in combination with the two reporter plasmids listed in S2 Table.

Strain	Genotype	Reference
*S.*Tm^{wt}	*SL1344, *wt* (SB300; SmR)	1
*S.*Tm$^{\Delta invG}$	SL1344, Δ*invG* (SB161; SmR)	2

Table S2. Plasmids used in this study.

Plasmid	Description	Reference
pM975	p*ssaG*-GFP (SPI2-dependent, vacuolar)	3,4
p*uhpT*-GFP	p*uhpT*-GFP (cytosolic)	5,6

Table S3. Primers used for RT-qPCR in this study. Concentrations of primers were 200 nM.

Target	Name	Sequence	Efficiency (%)
Cpa3	Cpa3 F	GAAAGTTGCAAGGATTGCCAC	99
Cpa3	Cpa3 R	TTGTGGATGCTATTGGGCCGT	99
Mcpt1	Mcpt1 F	TCCTGATGGCACTTCTCTTGC	107
Mcpt1	Mcpt1 R	TCCACTACAGTGTGCAGCAGT	107
Mcpt2	Mcpt2 F	TGTGTGATAGTGTGGCCCATG	112
Mcpt2	Mcpt2 R	TCTGACTCAGGCTGGTTAGGC	112
Mcpt4	Mcpt4 F	TAGACCACATTCTCGCCCTTA	111
Mcpt4	Mcpt4 R	GGATTCTGTCTTGCTCACATCA	111
Mcpt5	Mcpt5 F	TCCCACTCTCTGCCAACTTCA	103
Mcpt5	Mcpt5 R	TGGCTCATTCACGTTTGTTCT	103
Mcpt6	Mcpt6 F	TGGCATGCTGTGTGCTGGAAA	105
Mcpt6	Mcpt6 R	AGGTACCCTTCACTTTGCAGA	105
Mrgprb2	Mrgprb2 F	ATCAAGAATCTAAGCACCTCAGC	117
Mrgprb2	Mrgprb2 R	GAAAGCAAAATCATGGCTTGGT	117
Gata2	Gata2 F	CACCCCGCCGTATTGAATG	89
Gata2	Gata2 R	CCTGCGAGTCGAGATGGTTG	89
Il10	Il10 F	GCTCTTACTGACTGGCATGAG	115
Il10	Il10 R	CGCAGCTCTAGGAGCATGTG	115
Tlr4	Tlr4 F	ATGGCATGGCTTACACCACC	105
Tlr4	Tlr4 R	GAGGCCAATTTTGTCTCCACA	105
Tlr2	Tlr2 F	GCAAACGCTGTTCTGCTCAG	101
Tlr2	Tlr2 R	AGGCGTCTCCCTCTATTGTATT	101
Gapdh	Gapdh F	TGTGTCCGTCGTGGATCTGA	95
Gapdh	Gapdh R	TTGCTGTTGAAGTCGCAGGAG	95

Table S4. Primary antibodies used in this study, with unique identifiers (RRID).

Antigen	RRID	Conjugate	Host	Catalogue no.	Clone	Provider	Dilution
Mcpt1	AB_10854869	NA	Mouse	14-5503-82	RF6.1	eBioscience	1:200
Mcpt2	NA	NA	Rabbit	NA	Polyclonal	In house	1:1000
Mcpt6	NA	NA	Rabbit	NA	Polyclonal	In house	1:1000
Salmonella O Antigen Factor 5	AB_3676217	NA	Rabbit	226601	Polyclonal	BD, Difco	1:200
CD16/32	AB_394657	NA	Rat	553142	2.4G2	BD Biosciences	1:1000
FceR1 alpha	AB_2573493	PE-Cyanine7	Armenian hamster	25-5898-82	MAR-1	eBioscience	1:200
CD117 (c-kit)	AB_2739664	BV421	Rat	566290	2B8	BD Biosciences	1:200

References

1. Hoiseth, S. K. & Stocker, B. a. D. Aromatic-dependent Salmonella typhimurium are non-virulent and effective as live vaccines. *Nature* **291**, 238–239 (1981).
2. Kaniga, K., Bossio, J. C. & Galán, J. E. The Salmonella typhimurium invasion genes invF and invG encode homologues of the AraC and PulD family of proteins. *Mol. Microbiol.* **13**, 555–568 (1994).
3. Sellin, M. E. *et al.* Epithelium-Intrinsic NAIP/NLRC4 Inflammasome Drives Infected Enterocyte Expulsion to Restrict Salmonella Replication in the Intestinal Mucosa. *Cell Host Microbe* **16**, 237–248 (2014).
4. Hapfelmeier, S. *et al.* The Salmonella Pathogenicity Island (SPI)-2 and SPI-1 Type III Secretion Systems Allow Salmonella Serovar typhimurium to Trigger Colitis via MyD88-Dependent and MyD88-Independent Mechanisms1. *J. Immunol.* **174**, 1675–1685 (2005).
5. Hausmann, A. *et al.* Intestinal epithelial NAIP/NLRC4 restricts systemic dissemination of the adapted pathogen Salmonella Typhimurium due to site-specific bacterial PAMP expression. *Mucosal Immunol.* **13**, 530–544 (2020).
6. Geiser, P. *et al.* Determinants of divergent Salmonella and Shigella epithelial colonization strategies resolved in human enteroids and colonoids. *mBio* **0**, e00911-25 (2025).
