# Supplementary figures and images for: Mast Cell Phenotypic Heterogeneity Impacts the Interplay with Pathogenic Salmonella Typhimurium Bacteria
Source: Eur J Immunol. 2025 Aug 21;55(8):e70040. doi: 10.1002/eji.70040 (PMC12369454; doi:10.1002/eji.70040)

Figure 1 D

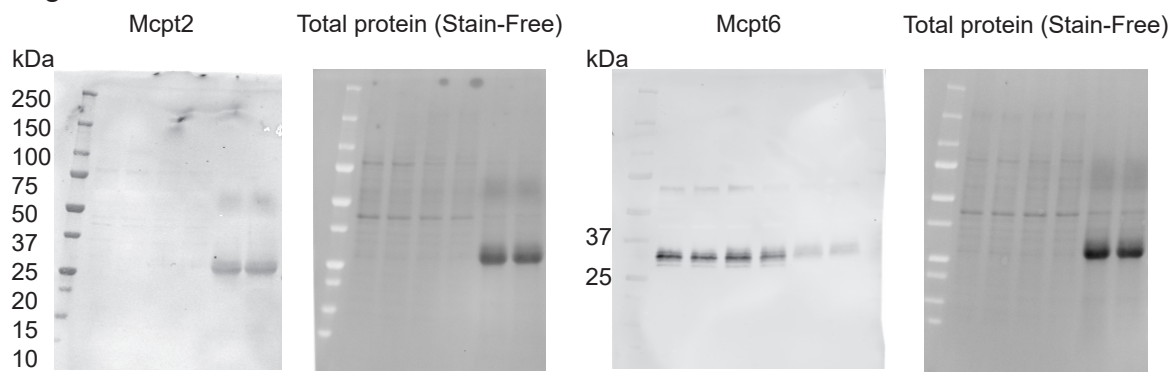

Figure S4 G

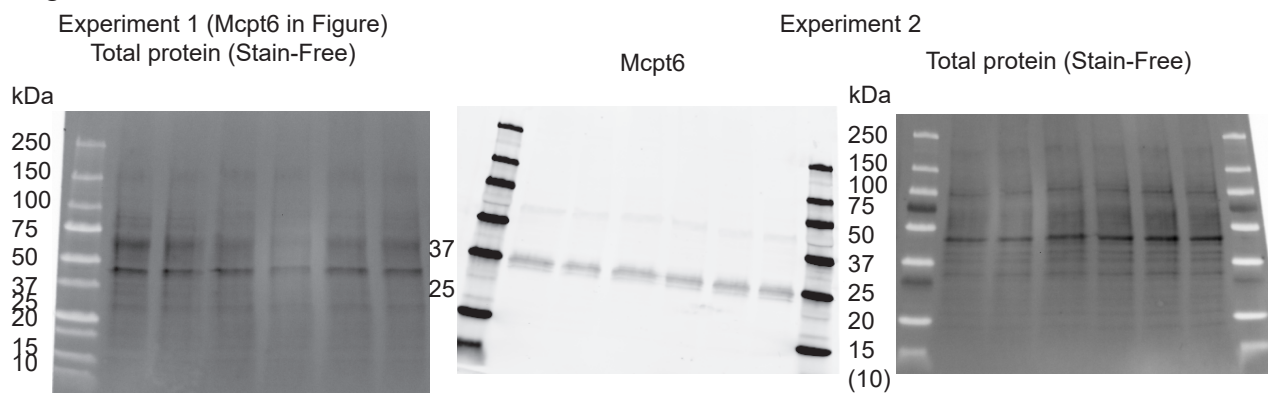

Figure S5 C

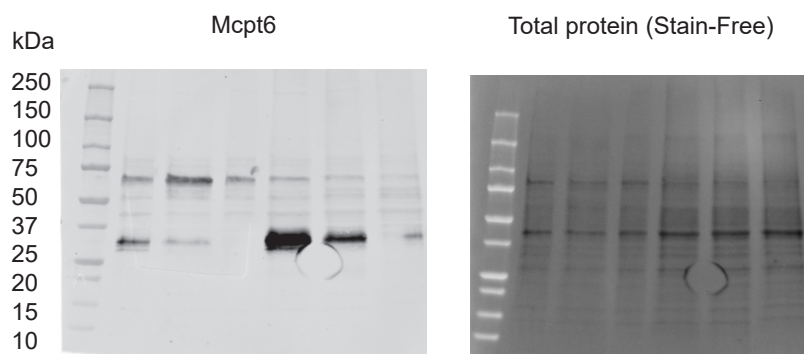

Figure S6 D

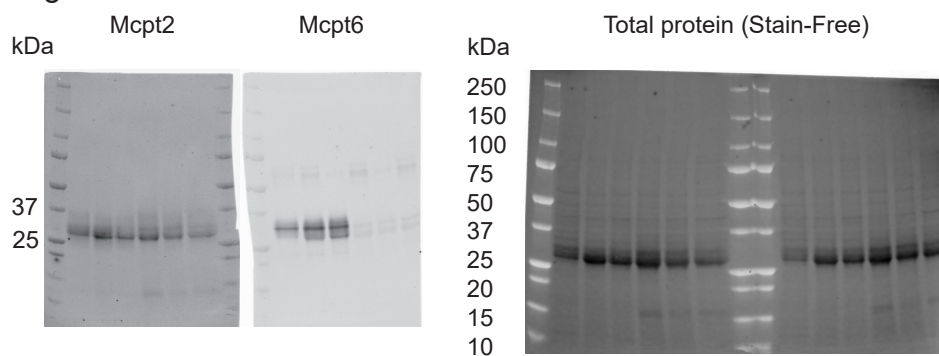

Supplement: Supplementary file 2 — Supporting File 2: eji70040‐sup‐0002‐SuppMat.pdf. [file EJI-55-e70040-s002.pdf]
